# Recent Advances in Voltammetry

**DOI:** 10.1002/open.201500042

**Published:** 2015-05-20

**Authors:** Christopher Batchelor-McAuley, Enno Kätelhön, Edward O Barnes, Richard G Compton, Eduardo Laborda, Angela Molina

**Affiliations:** a Department of Chemistry, Physical and Theoretical Chemistry Laboratory, University of OxfordSouth Parks Road, Oxford, OX1 3QZ, UK; b Departamento de Química Física, Facultad de Química, Regional Campus of International Excellence ‘Campus Mare Nostrum’, Universidad de Murcia30100, Murcia, Spain

**Keywords:** cyclic voltammetry, electrochemistry, electron transfer, kinetics, mass transport, nanoparticle electrochemistry, stripping voltammetry

## Abstract

Recent progress in the theory and practice of voltammetry is surveyed and evaluated. The transformation over the last decade of the level of modelling and simulation of experiments has realised major advances such that electrochemical techniques can be fully developed and applied to real chemical problems of distinct complexity. This review focuses on the topic areas of: multistep electrochemical processes, voltammetry in ionic liquids, the development and interpretation of theories of electron transfer (Butler–Volmer and Marcus–Hush), advances in voltammetric pulse techniques, stochastic random walk models of diffusion, the influence of migration under conditions of low support, voltammetry at rough and porous electrodes, and nanoparticle electrochemistry. The review of the latter field encompasses both the study of nanoparticle-modified electrodes, including stripping voltammetry and the new technique of ‘nano-impacts’.

## Introduction

The last decade or so has realised remarkable progress in the area of voltammetry—the interrogation of an electrode reaction by means of exploring, under dynamic conditions, the current-voltage characteristics of the process of interest so as to reveal, at one level, kinetic and mechanistic detail and, at another, more fundamental level, the validity or otherwise of theories of electron transfer.

Voltammetric measurements are easily (and cheaply!) carried out, and data rapidly accumulated. However, interpretation of the latter is often challenging, even to the well initiated especially if quantitative information is sought. In particular, until relatively recently, data analysis required the use of analytical equations confined by mathematical necessity to ‘model’ (or ‘toy’) systems under well-defined conditions of transport, electrode kinetics, and mechanism. As such, the area was often limited to the study of experimental systems which were amenable to data analysis rather than being driven by the physico-chemical interest of the system. The switch from this to real systems has been triggered by the ability to accurately simulate the voltammetric problems dictated and driven by chemistry rather than constrained by what is theoretically possible. The origins of this essential switch lies in the pioneering work of Rudolph^1^ and the subsequent commercialisation of his software in the form of the package DIGISIM.^2^ This initially provided the first general basis of the modelling of ‘nonstandard’ linear diffusion problems, subsequently developed to more complex problems, but above all providing the impetus for the physical electrochemist to model his or her own systems.^3^ It is the rigorous comparison of theoretical and experimental results^3^,^4^ that underpins the approach advocated in this review, highlighting how such methods enable greater physical insights into the dynamics of complex systems and the underlying operative chemistry.

This article surveys the progress made, not only in simulation, but also in analytical theory, and comprises seven main subject areas of interest. First (Section 1), the use of the Butler–Volmer equation for modelling electrochemical reactions is considered, specifically in terms of its application to multistep processes; this area is then further developed through consideration of the voltammetric response of electroactive species in ionic liquids. Ionic liquids are of both theoretical and practical interest due to their large electrochemical windows and the commonly observed altered chemical reactivity. Second (Section 2), the physical validity of the Butler–Volmer equation is questioned, and the development of the Marcus–Hush theory of electron transfer is probed from both a theoretical and experimental standpoint. Importantly, the presented ‘asymmetric Marcus–Hush’ theory enables physical reinterpretation of the Butler–Volmer equation which, in many cases, helps to validate its continued and historical use.

Recent voltammetric studies of the theories of electron transfer have, in a number of cases, been aided and facilitated by the use of pulse techniques. Moreover, such pulse techniques are also of distinct importance in the broad area of electro-analysis. Consequently, Section 3 of this review is dedicated to the theory of these often complex but highly important techniques. In particular ‘differential double pulse voltammetry′, ‘additive differential pulse voltammetry′, ‘reverse pulse voltammetry’, and ‘square-wave voltammetry’ are considered; a brief discussion of staircase cyclic voltammetry is also provided. In combination with this work, the concept of the diffusion layer is advanced, enabling insight into the concentration profile of electroactive species at the interface and diffusive mass transport as a whole. Following from this, Sections 4 and 5 specifically continue in the development of ideas surrounding mass transport. First, Section 4 reviews developments in the use and modelling of diffusive random walks. Such theoretical models are invaluable for the interpretation of newly developing stochastic single-molecule and single-nanoparticle techniques, facilitating new insights into mechanisms on the nanoscale. Second, Section 5 looks at the influence of migration upon the voltammetric response of electroactive species under conditions of low support. Beyond being a hindrance, low concentrations of supporting electrolyte can greatly facilitate electrochemical investigations, allowing mechanistic insights to be gained from the differing charges of the intermediates and their subsequent interaction with the electric field, as described by the Nernst–Planck–Poisson system of equations.

Having covered mass transport in relative detail, Section 6 considers the influence of altered electrode morphology upon the voltammetric response, where the local surface structure (rough, porous, etc.) serves to alter the diffusion regime local to the interface. The results of this section are of utmost importance for defining and evidencing *authentic* electrocatalysis. Finally, in light of the above discussion, Section 7 turns to consider nanoparticle electrochemistry. The section discusses nanoparticle-modified electrodes and the expanding field of stochastic ‘nano-impact’ experiments. Systems in which the nanoparticles mediate an electrochemical process are considered, as is the direct oxidation or reduction of the nanoparticles.

Although this review encompasses a very significant body of the work available within the literature, the areas highlighted are necessarily selective and focus upon fields deemed to be of particular contemporary importance; however, notable absences include both AC voltammetry^5^ and hydrodynamic^6^ techniques, amongst others.

## Section 1: Advancing Butler–Volmer Theory

### 1.1 Multistep electrode processes

Having performed a voltammetric experiment, observed a voltammetric wave, and correlated its electrochemical presence to a given redox species in solution, the next common scientific line of enquiry is to try and discern the operative electrochemical mechanism. A full review of this area of study is beyond the scope of the current text, and the interested reader is directed towards the seminal lecture series by Savéant^7^ and the in depth reviews by Evans.^8^ However, with the recent publication of the IUPAC recommendations on the transfer coefficient, the area of multistep electrode processes warrants brief attention.

The transfer coefficient (*α*) is now defined as;^9^

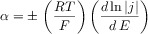
1

where the flux *j* has been “corrected for any changes in the reactant concentration at the electrode surface with respect to its bulk value”. The sign in Equation 1 depends upon whether the reaction is anodic or cathodic. This IUPAC definition of the transfer coefficient usefully and deliberately does not presuppose anything about the operative electrode reaction mechanism. Mass-transport corrections to attain the flux *j* relative to its bulk value is relatively facile for steady-state voltammetry.^10^ However, for macroelectrode cyclic voltammetry, complete extraction of this information is more involved, but, as demonstrated by Henstridge and Compton, certainly still obtainable.^11^ In most literature, experimental measurement of the transfer coefficient from macroelectrode voltammetry is limited to assessment of the Tafel slope at low overpotentials where the concentration of the reactant at the electrochemical interface is not significantly altered from that of the bulk value. Note classical literature tends to quote the magnitude of the Tafel slope (*d E*/*d* log|*j*|) which is directly related to reciprocal of the transfer coefficient. Having experimentally measured the transfer coefficient, its interpretation provides one route by which the electrode mechanism may be elucidated, as will be discussed below.

For a one-electron redox process with unit stoichiometry as given by

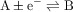
2

under reversible (quasi-equilibrium) conditions the transfer coefficient simply reflects the number of electrons transferred, which in this case is one. This value for the reversible case is generally referred to as an ‘apparent transfer coefficient’. The term ‘apparent’ is used to emphasise that the measured transfer coefficient does not reflect the underlying electron transfer kinetics, but originates directly from the system being under Nernstian control. Under irreversible conditions, the transfer coefficient takes a value between 0 and 1, but is commonly 0.5±0.2 for a one-electron process. As a note of caution, the transfer coefficient as measured from a voltammogram may be ‘artificially’ distorted due to a potentiostats application of staircase as opposed to a true analogue voltage ramp; this point is discussed in further detail in Section 3.4.

Use of the Butler–Volmer equation to model an electrochemical system implicitly assumes the transfer coefficient to be constant as a function of potential; deviations from such linear Tafel behavior have been experimentally evidenced. Moreover, the Butler–Volmer equation is periodically damned due to being ‘phenomenological’ in its description of electron-transfer, that is, it has no direct physical meaning. However, as will be expanded upon in Section 2 of this review, the Butler–Volmer equation can be better understood as often being a highly precise approximation of electron-transfer rates at low overpotentials and warrants its use due to its mathematical simplicity. Moreover, comparison of the Butler–Volmer theory with asymmetric Marcus–Hush theory allows a physical understanding of the transfer coefficient (see Section 2 for further details) in terms of the changing force constants in the redox reaction. Before moving on any further, it is highlighted that the terms ‘reversible’ and ‘irreversible’ when applied to voltammetry refer to the magnitude of the electron transfer rate relative to the prevailing rate of mass transport to and from the electrochemical interface.^12^ This is an important point that tacitly underpins much of the work discussed within this body of text. It is also a general lack of insight into this problem that regularly leads to misinterpreted and misreported results within the literature (see Section 7 for further discussion).

One of the principal points in the IUPAC recommendation is that the simultaneous transfer of two or more electrons is highly unlikely. Consequently, mechanistic interpretation of the transfer coefficient must be done in the light of this fact!^9^ At this stage, the simplest multistep electrochemical process is considered: that of two sequential reversible one-electron transfers (an *E*_rev_*E*_rev_ reaction in Testa and Reinmuth notation^13^) as described by

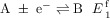
3

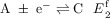
4

In the absence of coupled homogeneous kinetics the second electron transfer is likely to be less favorable purely due to coulombic repulsion.^8^ However, the relative potentials at which these two redox processes occur is highly solvent and electrolyte dependent;^14^ notably hydrogen bonding can have a profound influence.^15^ Experimentally, this situation is well exemplified by quinone reductions. In non-aqueous systems two one-electron waves are observed; the addition of water serves to compress the potential difference between the two waves until they merge to yield one voltammetric wave. However, in aqueous media (at high pH), it has been experimentally demonstrated that the potential of the first and second electron transfers can be ‘tuned’ through ion-pairing.^16^ A consequence of the first and second electron transfer occurring at similar potentials is that, although only one voltammetric wave is observed, the peak height is suppressed, and the wave is broader than would be anticipated if the electrons were assumed to be transferred simultaneously.^17^ In such cases, the use of square-wave voltammetry is advisable for the precise measurement of the associated formal potentials.^18^

**Figure 1:**
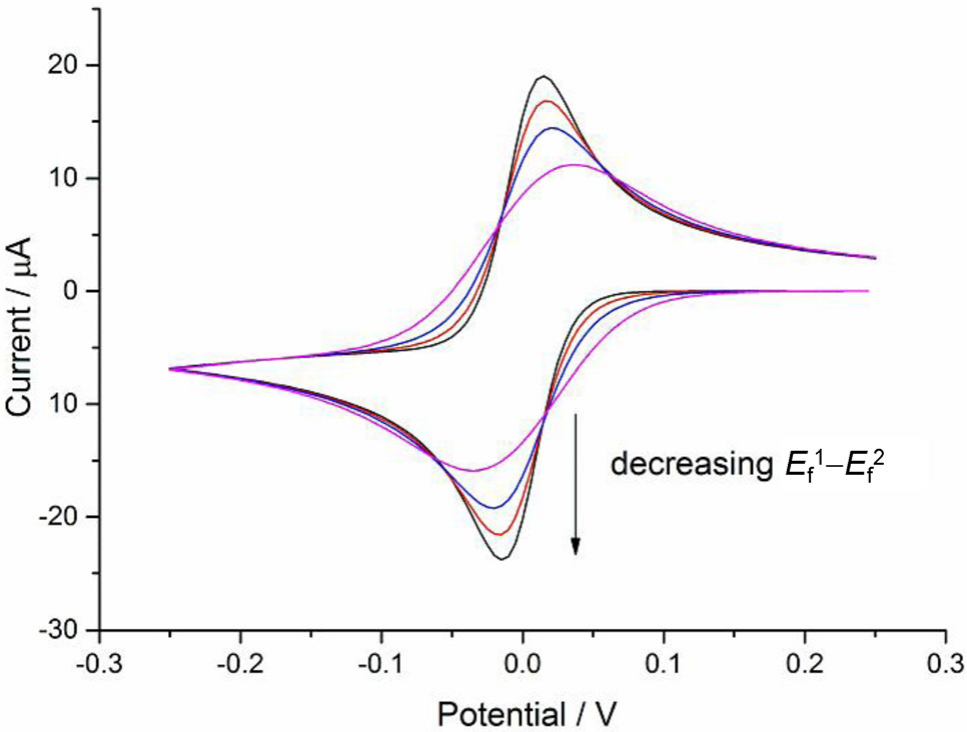
Simulated voltammetric response of a two-electron transfer as a function of the difference between the formal potentials for the two processes. *E*_f_^1^−*E*_f_^2^: +50 mV (magenta), 0 mV (blue), and −50 mV (red). The black line represents the hypothetical limiting case in which the two electrons are transferred simultaneously. Simulations performed using DigiSim; other simulation parameters: *v*=100 mV s^−1^, *D*=10^−5^ cm^2^ s^−1^, *r*_0_=1 mm.

The presence of coupled homogeneous chemical processes or structural changes in the molecular structure may lead to a situation known as potential ‘inversion’ where the second electron transfer is easier than the first, that is (*E*_f_^1^−*E*_f_^2^) is negative.^19^ Reportedly, a potential inversion of 400 mV is required such that ‘no’ intermediate is formed.^20^ Figure 1 depicts the simulated voltammetric response for a two-electron reduction as described by the mechanism above. Also shown is the predicted voltammetric response for the hypothetical situation in which two electrons are transferred simultaneously. This result clearly demonstrates how even for this simplest multistep process, the corresponding voltammetric wave shape can vary significantly when the two respective formal potentials are comparable in magnitude, a situation which is commonly encountered. Two important insights should be taken from this: first, a potential inversion of greater than 100 mV is required for the peak current of the stepwise two-electron voltammetric response to be within 3 % (i.e. within experimental error) of that obtained for the ‘simultaneous’ case. Second, a common route to determining the diffusion coefficient of an analyte is to measure the respective peak current as a function of scan rate, where the peak current is proportional to the square root of the diffusion coefficient. In the case of a multistep process, as highlighted by Figure 1, this may lead to a significant error due to the sensitivity of the peak current to the formal potentials of the electron transfers.^21^

As a secondary example, the case where an electron transfer is coupled to a homogeneous reaction as given by the mechanism:

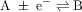
5

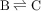
6

is considered. This scheme is known as an ‘EC reaction’ where E signifies an interfacial redox process and C is a coupled homogeneous reaction. For the case in which the electrochemical step is reversible, as the chemical step C is made more thermodynamically favorable (assuming it is not kinetically hindered), the potential required for the redox process decreases in magnitude, with a corresponding loss of the voltammetric back peak. As the chemical step becomes more highly driven, the redox wave shifts to a potential at which the rate of electron transfer becomes the rate-determining step (note the rate of electron transfer decreases exponentially, in accordance with the Butler–Volmer equation). Hence a ‘reversible’ electron transfer process (i.e. one which has a high *k*^0^) may appear electrochemically irreversible when the product is consumed by a highly driven chemical step.^22^

It can be seen from the above two examples that the complexity of an electron transfer reaction rapidly increases with the number of steps, and hence such systems are best understood through simulation (further discussion of multistep electrochemical processes can be found in the work of Batchelor–McAuley and Compton^21^). This is especially true for situations involving proton transfer where a multitude of possible mechanistic routes are possible, and the exact pathway (or pathways) taken will depend strongly upon the pH, the electrode potential, the pK_a_ value associated with the reactants, intermediates, and products, and the electron-transfer kinetics of individual steps.^17^ Related to this is the investigation of systems in which the proton and electron are transferred simultaneously, a reaction which is possibly of distinct importance in biology.^23^

Although full understanding of an electrochemical process is best achieved through simulation, the rate-determining step of a multistep mechanism may be readily assessed by experimental measurement of the Tafel slope, noting that the magnitude of the Tafel slope may also vary as a function of scan rate due to the presence and influence of coupled homogeneous kinetics.^24^ The Tafel slope should be interpreted as being equal to −(*n*′+*α*_RDS_)*F*/*RT* (for a cathodic process) where *n*′ is the number of electrons transferred before the rate-determining step, and *α*_RDS_ is the transfer coefficient of the rate-determining electron transfer.^21^ Noting that again, in accordance with IUPAC, assuming that the Tafel slope is equal to −(*αn*)*F*/*RT*, where *n* is the total number of electrons transferred is invalid and outdated as it implies the possibility of multiple electrons being transferred simultaneously. A problem arises upon recognition that many older analytical expressions used for interpreting voltammograms, which are still regularly used, are in error—one prime example being the use of the variation of the peak potential of a surface-bound redox wave as a function of scan rate to extract kinetic and mechanistic information.^25^ In this model, the peak position is related to *αn*; hence, use of such expressions for analysis of the operative mechanism will be in error.

As commented above in reference to electrochemical reversibility, voltammetry is inherently the study of interfacial processes; consequently, understanding of a systems response requires an understanding of the prevailing mass-transport regime and its effect. An example of this for multistep processes relates to the influence of an electrodes size. On decreasing the dimensions of an electrode, that is from macro to micro, the mass transport to and from the surface becomes more efficient.^26^ This is akin to increasing the rotation rate of a rotating disk electrode, however far higher mass-transport rates are attainable with the use of microelectrodes.^27^ The decrease in the electrode size results in the electron transfer process becoming more irreversible and increases the probability of intermediates being released. To more succinctly restate this, on changing the electrode size the overall electrochemical mechanism may be altered, resulting in the possible release of ‘higher’-energy intermediates. One example of such a situation is found with oxygen reduction at silver surfaces. At a macroelectrode the process is found to, on average, involve the transfer of 3.3 electrons to each oxygen molecule.^28^ This corresponds to roughly one in three oxygen molecules undergoing only a two-electron reduction to hydrogen peroxide before release from the electrode, as opposed to the full four-electron reduction. However, decrease of the size of the electrode leads to an increase in this ratio, such that the probability of hydrogen peroxide production is increased, where on the nanoscale, almost all formed hydrogen peroxide is released prior to further reduction.^28^ This insight that reactivity may change on the nanoscale solely due to the altered mass transport (i.e. even without considering plausibly altered nanoparticle thermodynamics or the expression of higher-order crystal facets) has two implications: first, it implies a limitation on the efficiency of the use of nanoparticles for catalysis in industrially relevant processes.^25^ Secondly, and perhaps more importantly, the changed reactivity on the nanoscale arising from the altered mass-transport regime may have wide-ranging implications for the nanotoxicity of these materials towards biological systems.^29^ It is this changed reactivity at diffusionally isolated particles that highlights one of the driving forces for wishing to study reactions at individual nanoparticles, a subject that will be focused upon more within the final section of this review.

The following section focusses on the simulation and understanding of voltammetry in ionic liquids, a medium in which the mass-transport rates are appreciably slower than found for common aqueous systems.

### 1.2 Voltammetry in ionic liquids

The last decade has seen an explosion of electrochemical interest in the use of room-temperature ionic liquids (RTILs): liquids composed entirely of ions which only solidify at temperatures well below ambient.^30^ This is partly because they offer significant advantages for some applications, most notably in energy transformation technology^31^ and in gas sensing,^32^ but also since they challenge existing theories of electron transfer^33^ and interfacial structure.^34^ Progress in the area has been regularly reviewed.^30^,^35^ This section focuses on the altered voltammetry seen in RTIL media.

From an electrochemical perspective, RTILs offer some important contrasts with conventional solvents such as water, acetonitrile, THF, etc.. First, the potential window displayed, defined by the onset of cathodic and anodic solvent decomposition, is unusually wide and, for rigorously dried solvents, can extend to as much as 5 or 6 V.^36^ This reflects the stability towards oxidation and reduction of the component ions which are generally a bulky organic cation and a small inorganic anion such that the size mismatch discourages crystallisation except at unusually low temperatures. Indeed the use of the same materials as supporting electrolyte in conventional solvents has been advocated, reflecting the intrinsic inertness of the ions.^37^

A second significant difference lies in the observation^30^ that many ionic liquids have viscosities which are larger—often an order of magnitude greater—than conventional molecular organic solvents. This is reflected in the magnitude of the diffusion coefficient of the solutes which, except for rather small sized molecules such as O_2_^38^ or H_2_S,^39^ generally reflect the Stokes–Einstein equation, with diffusion coefficients scaling inversely with the viscosity.^40^ The much reduced rates of diffusion in RTIL media have the very important consequence that the transition from linear to fully convergent diffusion as observed at microelectrodes occurs at quite different (much lower) voltage scan rates than are familiar to electrochemists operating in aqueous or non-aqueous media.^41^ As a result, it is quite common to see peak-shaped rather than sigmoidal current voltage curves when using microelectrodes in ionic liquid media, and true steady-state diffusion limited currents can be difficult to observe unless unusually if not pathologically slow scan rates are deployed (under which conditions other factors such as slow adsorption or coupled kinetics may undesirably kick in). It follows that the extraction of kinetic and transport parameters from the voltammetry requires the numerical simulation^41^ of the voltammetry rather than the application of the simple analytical equations derived for pure linear diffusion (Randles–Ševčík equations) and for pure convergent diffusion at a microdisc (*I*=4*nFDCr*, where *n* is the number of electrons transferred, *F* is the Faraday constant, *D* is the diffusion coefficient, *C* is the analyte concentration, and *r* is the radius of the electrode). Moreover, the fitting of such simulations is challenging, but can be helpfully simplified if the diffusion coefficients of the reactant, A, and product, B, are determined for:

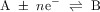
7

independently of the voltammetry, ideally using potential-step or double-step chronoamperometry.^42^

A third but related issue is that the diffusion coefficients of the analytes in RTIL media can be quite sensitive to the presence of dissolved gases not least because of the high solubilities (∼m) of species such as H_2_S or CO_2_ in many RTILs, but also because the dissolved gases significantly perturb the solvent structure and hence the transport of the other solutes.^43^

A fourth issue relating to the different voltammetry in RTIL media as compared to molecular solvents is that the common approximation of assuming equal diffusion coefficients for most or all species involved in an electrode reaction is usually adequate for the quantitative simulation of the voltammetry; this approximation holds much less well for RTIL media. The reason for this is that because of the ionic nature of the solvent, the transport properties are sensitive not only to the solute size (Stokes-Einstein equation) but also to the solute charge.^44^ One extreme example is the one-electron reduction of oxygen,

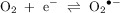
8

in the RTIL hexyltriethylammonium bis(trifluoromethyl)sulfonyl imide, where at 25 °C

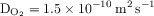


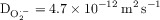


which gives a ratio of diffusion coefficient of over 30!^45^ The consequence of this marked difference is that the voltammetry leads to curious current-voltage response in which a microdisc electrode steady-state voltammogram is seen for the forward scan, corresponding to the faster diffusing O_2_ reduction, whereas in the reverse scan a peak is observed for the reoxidation of O_2_^.−^ because the slowness of its diffusion leads to the accumulation of O_2_^.−^ near the electrode surface, and the transport contains a significant component of linear diffusion.^45^

The discussion above has focused on measurements made in RTIL media using microelectrodes, which are a preferred methodology for such investigations.^32^,^46^ This is because the deployment of microelectrodes facilitates the use of small volumes (∼10 μL) of solvent, which is important in the RTIL area since it is vital to properly dry the solvent and to ensure that they are water free. The drying of RTILs is readily undertaken using a T-cell arrangement^32^,^46^,^47^ in which the solvent can be exposed to vacuum and rigorously dried before voltammetric study. Any residual water will greatly reduce the electrochemical window36b and markedly alter the diffusion coefficients of the solutes.^46^ The drying of larger quantities of solvent is a slow process because of the need for the water to diffuse to the liquid surface and evaporate; as observed diffusion in RTILs can be a very slow process, and so the purification of the much larger volumes required for macroelectrode experiments would be time-consuming and possibly incomplete. Indeed the lack of reproducibility of simple data such as diffusion coefficients (even of ‘model’ compounds such as ferrocene), in early work in the field probably reflects the different composition of the solvent used in terms of dissolved water and gases.

Finally, we consider whether Butler–Volmer theory is applicable in RTIL media noting the near-ubiquitous success claimed for the phenomenological approach in molecular solvents. The first consideration is to note that the slowed diffusion in RTILs promotes the apparent electrochemical reversibility of many redox couples. In order for the voltammetry to reveal quasi- or irreversible electrode kinetic behavior, it is required that the studied rate constant must fulfill *k*^0^<*m*_T_, where *m*_T_ (cm s^−1^) is the mass-transport coefficient of the electrode. Typically this is approximated by *m*_T_∼*D*/*r*, where *r* is the electrode radius, so that even with microelectrodes it can be challenging to extract electrochemical rate constants from the voltammetric data. It follows that measurements made using macroelectrodes in RTIL are unlikely to give reliable data especially since it is noted that the conductivity of many ionic liquids is similar to that of conventional organic solvents (DMF, CH_3_CN, THF, etc.) containing about 0.1 m supporting electrolyte so that macroelectrode voltammetry in RTILs is also typically as distorted by ohmic losses as is voltammetry in organic media.

Extensive modeling of a wide diversity of voltammetric systems has been undertaken using small microelectrodes in order to provide a better possibility of extracting kinetic parameters. Systems studied include O_2_/O_2_^.−^,^45^ Br^−^/Br_2_,^48^ nitrobenzenes,^49^ aryl amines,^50^ NO_2_/NO_2_^−^/NO_2_^+^,^51^ I^−^/I_2,_^52^ aromatic diamines,^53^ arenes,^54^ Li/Li^+^,^55^ benzoquinone,^56^ hydroquinone,^57^ and H^+^/H_2_.^58^ In many cases the values of *k*^0^ obtained correspond to quasi-reversible behaviour and, as such, do not provide a perfect test of the validity of Butler–Volmer kinetics since the behaviour is approximately Nernstian. To restate this, under reversible (Nernstian) conditions, no information regarding the kinetics of the electron transfer process may be inferred from a voltammetric experiment. Hence, as a system tends towards reversibility, obtaining unambiguous results evidencing the validity or otherwise of the applicability of the Butler–Volmer equation becomes inherently more challenging. However, in some cases, notably the I^−^/I_2_ system,^52^ the oxidation of hydroquinone,^57^ Li/Li^+^,^55^ and the H^+^/H_2_ system^58^ there is clear electrochemical irreversibility, and the accuracy of Butler–Volmer kinetics in reproducing observed experimental behavior is excellent. Note that the follow-up chemistry in these systems ‘promotes’ the irreversibility of the system and, as such, multistep processes may be preferred for studying electron transfer in RTILs.^59^

Very recently,^60^ an attempt has been made to apply Marcus–Hush theory to ionic liquids focusing on the O_2_/O_2_^.−^ couple. Solvent reorganisation energies around 0.4–0.5 eV were found and attributed to inner sphere reorganisation with a negligible contribution from solvent reorganisation.

In summary, quantitative voltammetry in RTILs present special challenges, but with the aid of numerical simulation, quantitative understanding of the both kinetics and mechanism is possible. The following section looks at recent advances in the development of models of electron transfer kinetics.

## Section 2: Challenging Butler–Volmer Theory

In recent years, a renewed interest in the suitability of the available kinetic models for heterogeneous electron transfer reactions has led to critical assessment^61^ of the most well-established approaches: the Butler–Volmer (BV)^62^ and the Marcus–Hush (MH)^63^ models.

The BV formalism has been preferred over the years (and still in the present) due to its simplicity and satisfactory description of the electrode kinetics of many systems with three fitting parameters: the standard heterogeneous rate constant (*k*^0^), the transfer coefficient (*α*) and the formal potential (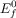). Thus, the rate constants are given by the following well-known expressions:

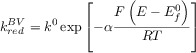
11

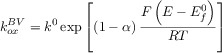
12

In spite of being successful in kinetic parameterisation of a great majority of redox systems, easy-to-implement, and computationally inexpensive, the BV expressions for the rate constants are empirical (but see below in connection with the discussion of asymmetric Marcus–Hush theory). Therefore, the adjustable parameters provide limited physical insight in terms of the nature of the electroactive molecules, the medium, and the electrode, and it is not possible to make predictions. Moreover, experimental deviations from the ever-increasing exponential variation of the rate constants with 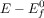
predicted by Equations 9 and 10 have been reported.^64^

The above limitations of BV calls for the use of more realistic models that enable us to fully describe the experimental data, as well as to connect the electron transfer kinetics with the nature of the system. With this aim, in recent years the applicability of the Marcus–Hush model has been theoretically and experimentally assessed via voltammetry in its symmetric64b and asymmetric^65^ versions.

### 2.1 The symmetric Marcus–Hush model (sMH)

The symmetric version of Marcus theory^66^ considers the parabolas describing the Gibbs energy of the reactants and products to be of equal curvature. The heterogeneous electrochemical reaction between a molecule and a metallic electrode involves transfer of charge from a discrete molecular energy level to a continuum of states (*ɛ*), with the energy levels in the electrode occupied according to the Fermi–Dirac distribution. This model leads to the following expressions for the heterogeneous rate constants:64b

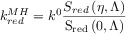
13

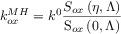
14

where 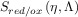
are integrals:

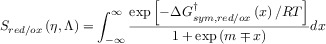
15

with 
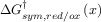
being the activation energy of the electro-reduction/oxidation process for each electronic level that according to the symmetric version of the Marcus theory is given by:

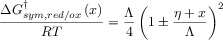
16

where 
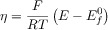
, 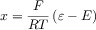, and Λ is the dimensionless reorganisation energy: 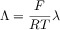. When two signs appear, the upper sign refers to reduction and the lower sign refers to oxidation. For the calculation of the integrals of the sMH formalism [Eq. (13)], numerical integration methods can be employed^67^ and analytical approximations have also been proposed to make the implementation of the sMH expressions easier.^68^

As with the BV model, sMH theory describes the electron transfer kinetics as a function of three adjustable parameters: the formal potential, the standard heterogeneous rate constant, and the reorganisation energy (*λ*). The latter corresponds to the energy required to distort the atomic configurations of the reactant molecule (inner-sphere component of *λ*) and its solvation shell (outer-sphere component) to those of the product in its equilibrium configuration. Therefore, *λ* enables us to rationalise the electrode kinetics in terms of the microscopic nature of the system such that the larger the structural and solvation changes as a consequence of the electron transfer, the larger the *λ* value and the slower the electrode kinetics.

### 2.3 The asymmetric Marcus–Hush model (aMH)

In the sMH model, the Gibbs energy parabolas are assumed to have the same curvature, which means that intramolecular vibrations and solvation are, on average, the same for the reduced and oxidised species. This may not hold as a general rule given the different charge of the reduced and oxidised species, and various theoretical approaches have been considered to overcome this limitation of the sMH formalism. Among them, the use of the asymmetric version of the Marcus theory has been recently applied to heterogeneous electron transfer processes by Compton et al.61a

**Figure 2:**
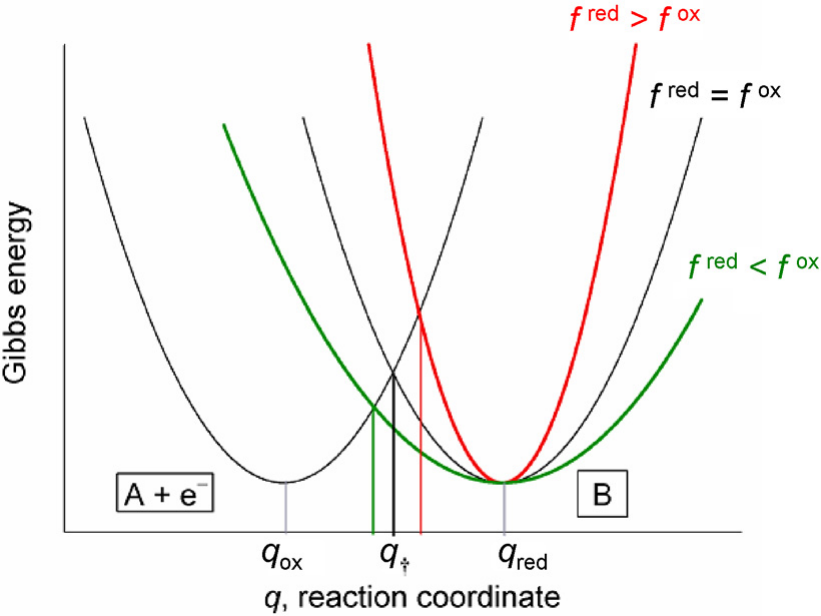
Schematic of the parabolic Gibbs energy curves as given by the asymmetric version of the Marcus theory.

As can be observed in Figure 2, different (vibrational and/or solvation) force constants result in Gibbs energy curves of different curvature and affect the value of the activation energy that, within the asymmetric Marcus theory, can be written as:65a-66

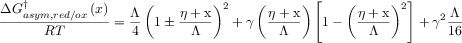
17

with the parameter *γ* being defined as:

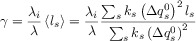
18

where *λ*_i_ is the inner-sphere reorganisation energy, 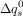
is the difference between the equilibrium values for the *s*-th normal mode coordinate of reactant and product, and *k*_s_ and *l*_s_ are symmetric and antisymmetric combinations of the force constants of the *s*-th mode of the oxidised (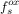) and reduced (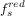) species:

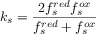
19

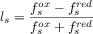
20

The values of the rate constants in the aMH model are calculated from the expressions presented in Section 2.1 by substituting Equation 15 into 13. In the numerical integration of Equation 13, the limits of the integral must be restricted to the *x*-range where the integrand value is significant, typically ±50.

From the definitions in Equations 16, 17, and 18, it is clear that the *γ* value relates to differences between the vibrational force constants of the electroactive species such that it takes a positive value when the force constants of the oxidised species are greater (on average), a negative value in the opposite situation, and *γ*=0 when the (average) force constants are equal. Note that the last particular case coincide with the symmetric MH model such that Equation 15 simplifies to 14 for *γ*=0.

In the derivation of Equation 15, only the first terms in the expansion of 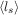
have been considered so that the aMH formalism above presented accounts for differences between the vibrational modes of the reduced and oxidised species with only one additional fitting parameter with respect to BV and sMH (γ, [Eq. (16)]). Higher order terms in 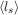
66 would be necessary when the force constants differ significantly (by a factor of more than 261a), which would make the model less general and more complex.

### 2.4 Voltammetric assessment of the BV and MH models

**Figure 3:**
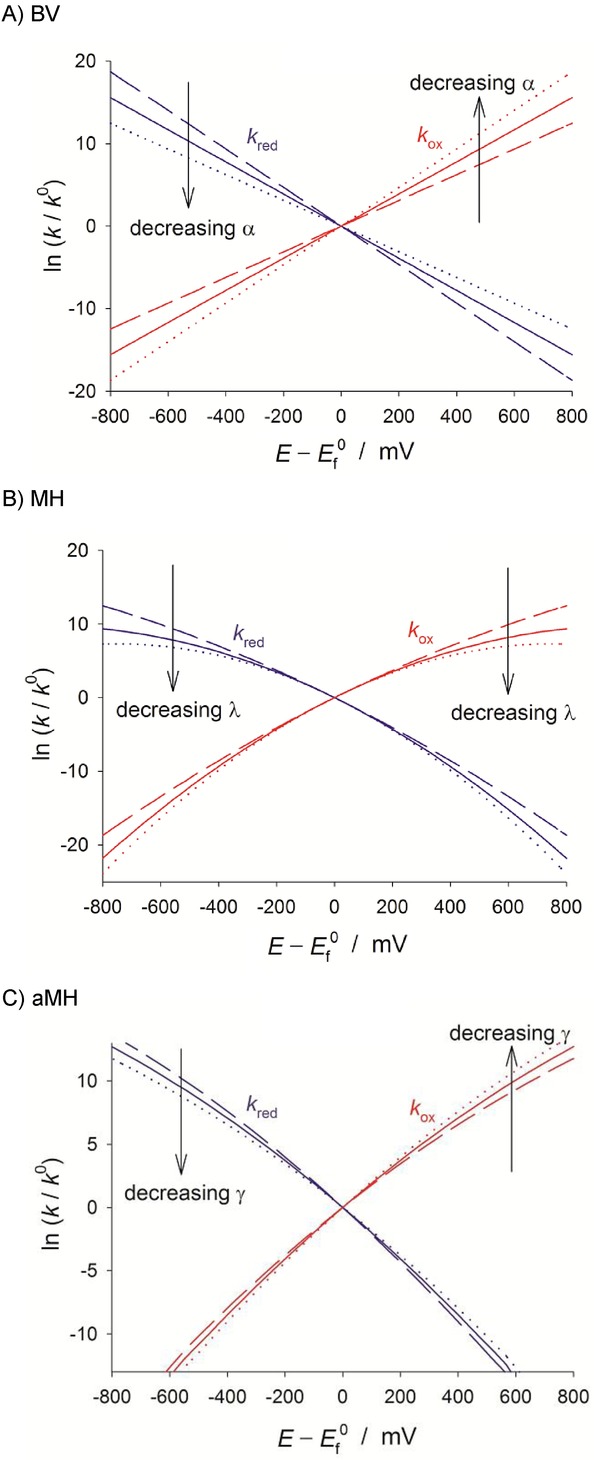
Variation of the reduction and oxidation rate constants with *E*−*E*_f_^0^ in the Butler—Volmer model (A), the Marcus—Hush models (B), and the asymmetric Marcus–Hush model (*λ*=2 eV) (C).

As shown in Figure 3 the different kinetic models predict different variations of the rate constants with the applied potential. Thus, whereas the reductive rate constant increases exponentially and continuously as 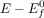
is more negative, in the MH models *k*_red_ shows a limiting value at large 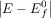
values, which is consistent with the curved Tafel plots and potential-dependent transfer coefficients reported in the literature.^64^ The divergence from the BV behaviour is more apparent for small λ-values and at large overpotentials (Figure 3 b).

Another key point to consider in Figure 3 is that, independently of the *λ*-value, the curves for the reduction and oxidation rate constants are symmetrical with respect to the axis 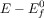
=0 in sMH, such that *k*_red_(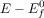)=*k*_ox_(−(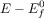). This reciprocity relation68a breaks down in the aMH model when γ≠0. Thus, when the force constants of the oxidised species are greater, *γ*>0, the cathodic branch is steeper than the anodic one, and the opposite is true for γ<0. Note that the *γ*-effect is more significant at large overpotentials and it is analogous to the effect of *α* in BV. Indeed, at low overpotentials, the *k*_red/ox_ values calculated from the aMH formalism tend to those obtained in BV with the following transfer coefficient:

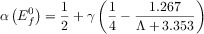
21

Thus, the case *α*<0.5 relates to force constants of the reduced species greater than those of the oxidised one (i.e., *γ*<0) and the opposite applies for *α*>0.5. This enables physical reinterpretation of the *α* data available from Butler–Volmer analysis, extending over many years.

**Figure 4:**
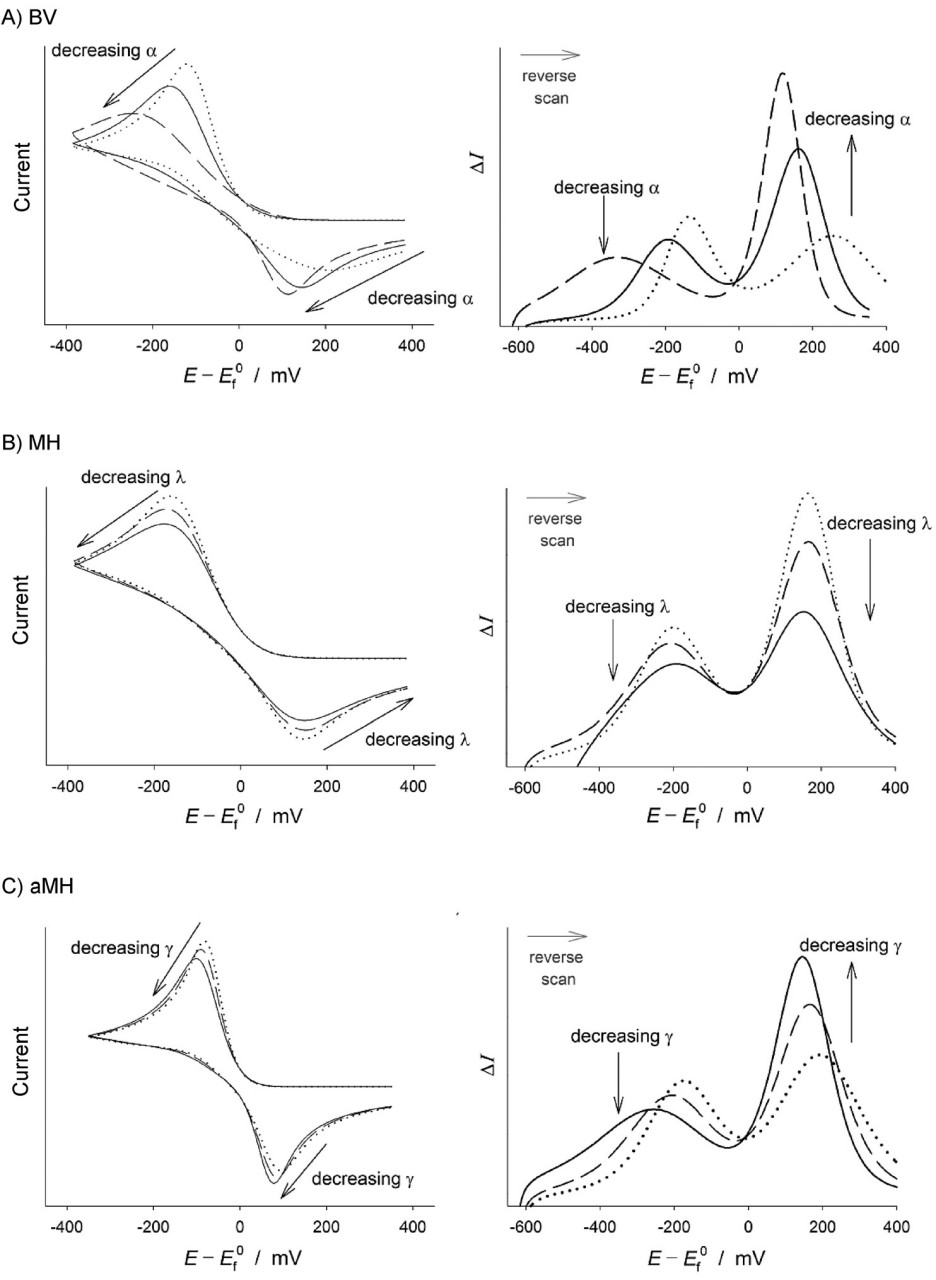
Influence of the kinetic parameters of the different kinetic models on the response of diffusional quasi-reversible and irreversible systems in cyclic voltammetry and reverse scan square wave voltammetry.

The effect of the asymmetric parameter *γ* on the voltammetric response is also analogous to that of *α* in BV. This is shown in Figure 4 for the response of diffusional systems in cyclic voltammetry and reverse scan square wave voltammetry under transient conditions. In the latter, as well as in the reverse scan of cyclic SWV, a cathodic peak and an anodic one can be observed on either side of the formal potential in the case of sluggish electron transfers.^69^ In both CV and SWV, the reorganisation energy affects the reductive and oxidative peaks of the voltammograms similarly (Figure 4 b), whereas the *γ*-value has an effect on the relative anodic/cathodic peak heights and the peak potentials. The reductive peak increases in height and shifts to less negative potentials as *γ* takes more positive values, as occurs in BV for *α*>0.5. This fact points out the greater flexibility of the aMH model for quantitative fitting of the voltammetry through the new kinetic parameter.

In summary, the kinetic formalisms discussed above predict different dependence of the reduction and oxidation rate constants with the applied potential, the divergence between them being more apparent for small values of the reorganisation energy and at large overpotentials. In order to point out such differences experimentally and assess the suitability of the different kinetic models, various electrochemical methods have been proposed and employed in the literature as an alternative (or complement) to cyclic voltammetry.^70^ The use of differential pulse voltammetries (namely, square wave voltammetry and differential multipulse voltammetry) has proven very insightful in revealing differences between the kinetic models^71^ as well as being very adequate for quantitative studies. Thus, due to the subtractive nature of these techniques, well-defined, peak-shaped responses are obtained and undesirable distortions associated with double layer charging and other possible background processes can be reduced. Differential pulse techniques in reverse or cyclic modes are of particular value given that, as shown in Figure 4, they enable simultaneous examination of the reduction and oxidation processes, which is essential to confirm the consistency of the kinetic parameters obtained from the fitting of experimental data.61e Also, the value of the large amplitude Fourier-transformed AC voltammetry has been examined.70b The analysis of the higher order harmonic responses and the frequency-dependence of the peak heights of the harmonics is predicted to be very powerful and sensitive in the study of the applicability of the different kinetic models and the extraction of kinetic parameters.

It is also worth highlighting that only the aMH model is compatible with the asymmetric, curved Tafel plots obtained experimentally for surface-bound and diffusional redox systems,^64^ as demonstrated in work by Henstridge et al.65b by the fitting of experimental data available in the literature.64c Other contrasting behaviours between the kinetic formalisms have been theoretically described and they potentially allow for critical evaluation of the models, though the experimental conditions necessary are challenging. Thus, the sMH and aMH models predict deviations from the Randles–Ševčík behaviour for irreversible processes.^72^ The experimental evidence of such deviations is not straightforward, particularly in the case of diffusional systems, given that it requires the study of systems with small reorganisation energy (unlikely in the case of slow kinetics) in a broad range of scan rates.

Another striking difference between the BV and the MH models is that in the latter, the limiting current (in single and double-step chronoamperometry, as well as at fast-flow channel electrodes70a) can be smaller than the mass-transport-controlled limit and depend upon the electrode kinetics.^61^,70a Again, this phenomenon is predicted to be more apparent for small values of the reorganisation energy. Given that this is in general associated with fast kinetics (i.e., large *k*^0^ values), special attention has been paid to the use of nanosize (including nanodiscs61d,61e and impacting nanoparticles^73^), nanogap,61b,61c and channel^74^ electrodes such that the enhanced mass transport shifts the kinetic-controlled voltammetric response away from 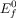. Thus, it is theoretically possible to observe kinetically-limited steady-state currents at large overpotentials in the above systems when the size of the electrode or the gap distance is reduced to the nanometer scale, though in practice this requires that the geometry of the electrode is accurately known and, in the case of electrodes of a few nanometers, to deal with double layer and nonclassical effects.

### 2.5 Experimental assessment of the kinetic models

A critical study of the models presented above has recently been undertaken by studying the voltammetric response of various solution-phase systems, including the one-electron reductions of 2-methyl-2-nitropropane, 1-nitropentane, 3-nitrophenolate, cyclooctatetraene, and europium(III), as well as the electro-oxidation of tetraphenylethylene.61a,65c As concluded from Figure 3, in order to observe differences between the BV, sMH, and aMH models, the experimental system must give a kinetically controlled current at appreciable overpotentials. This has been achieved by Compton et al. for electrode processes with *k*^0^≤0.02 cm s^−1^ by using microelectrodes of 25–50 μm radius, which also allows for the reduction of undesirable ohmic drop and capacitive effects. The use of nanosize61d,61e,^73^ or nanogap61b electrodes would be necessary for faster electron transfers, which presents difficulties in terms of electrode fabrication and characterisation as well as modeling of nonconventional effects.

The voltammetric response in different techniques (mainly cyclic and square wave voltammetries) of several one-electron transfer processes without chemical complications and under fully-supported conditions has been analyzed making use of the three kinetic models. The sMH model has been unable to fit the experimental voltammetry of systems with transfer coefficients notably different from 0.5 (as those chosen in the experimental studies), which is expected in light of the results discussed in Section 2.4. On the other hand, the BV and aMH formalisms yield satisfactory fittings of similar quality, with the correlation between the parameters *γ* and *α* above-mentioned being found experimentally, such that *α*-values different from 0.5 may be interpreted as an indicator of different force constants in the oxidised and reduced species. Note that such differences can also arise from the interactions with the solvent as theoretically demonstrated in Laborda et al.^75^ making use of the nonlinear Matyushov solvation model.^76^

According to all of the above, for solution-phase redox couples, the simpler, 3-parameter BV model can be recommended for the fitting of experimental data, complemented with the physical insights derived from the asymmetric Marcus model. On the other hand, the analysis of surface-bound redox couples should be performed using the asymmetric Marcus–Hush model, which is the only theoretical approach (among those considered here) compatible with all the experimental results reported in the literature.

## Section 3: Advances in Voltammetric Techniques

### 3.1 Double potential pulse techniques at microelectrodes

**Figure 5:**
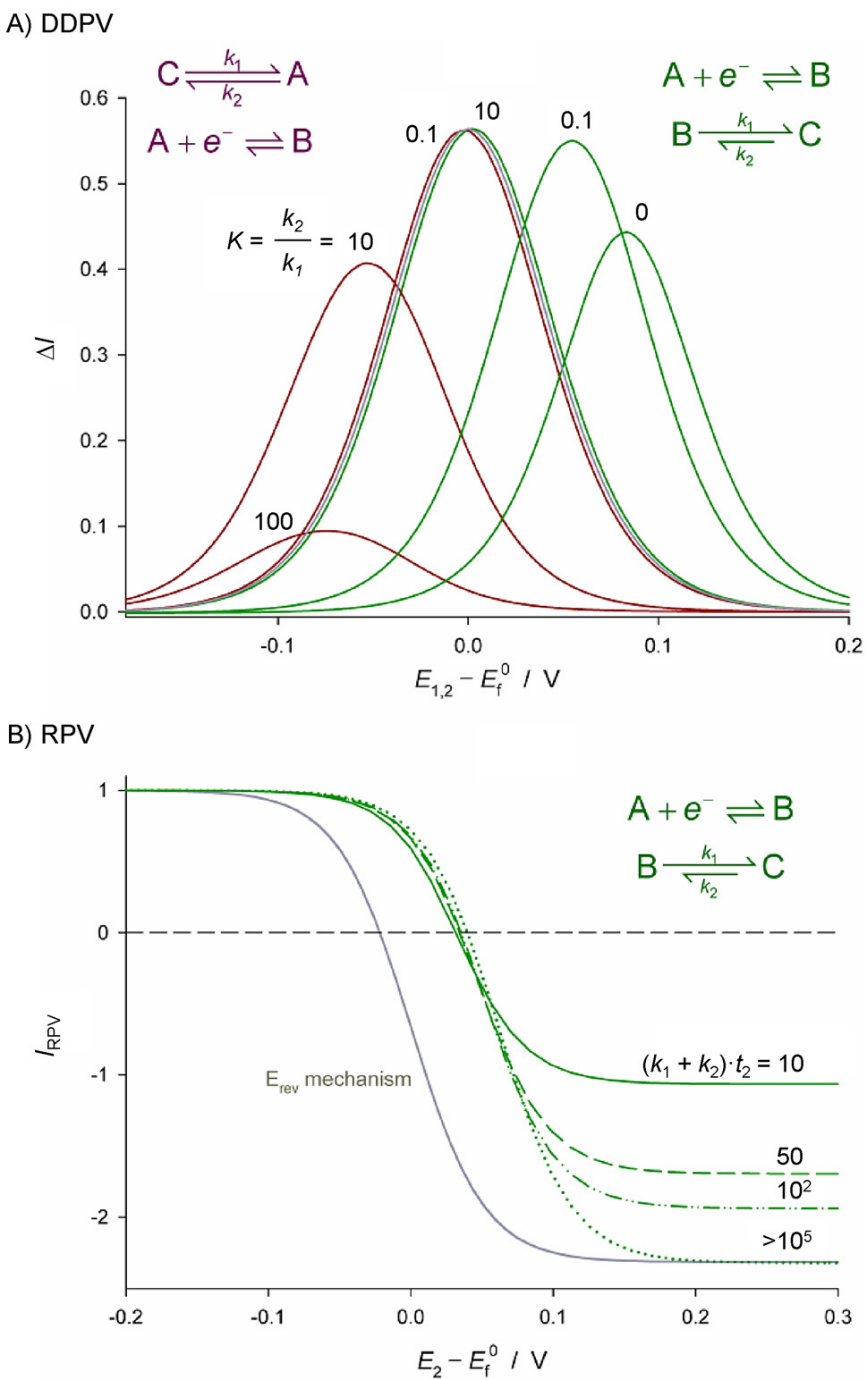
Illustration of the effects of the thermodynamics and kinetics of coupled homogeneous chemical reactions on differential double pulse voltammetry (DDPV)^[79a]^ (A) and reverse pulse voltammetry (RPV)^[80]^ (B). Grey solid lines correspond to a simple reversible E mechanism.

In recent years the use of double potential pulse techniques for the study of electrode kinetics and reaction mechanisms has been developed both theoretically and experimentally at microelectrodes.^77^ The combination of pulse techniques and small-sized electrodes offers important advantages in terms of accuracy as a result of the reduction of distorting effects (mainly ohmic drop and charging current),^26^,^78^ which leads to well-defined signals adequate for electrochemical studies even in media of low conductivity. With respect to electrode reactions complicated by coupled (electro)chemical processes (Figure 5), analytical theory for double pulse techniques at microelectrodes of different geometries has been developed for the study of the (pseudo)first-order CE,^79^ EC,^79^,^80^ catalytic^81^ and equilibrium square^82^ mechanisms as well as multistep electrode processes.^83^ Analytical expressions for one-electron transfer processes of solution-phase redox systems of any reversibility degree have also been deduced for double potential pulse techniques at (hemi)spherical microelectrodes. The reversibility criteria and methodologies for kinetic analysis are appropriate for other microelectrode geometries and will be discussed in the following sections.

#### 3.1.1 Differential double pulse voltammetry (DDPV) and additive differential pulse voltammetry (ADPV)

**Figure 6:**
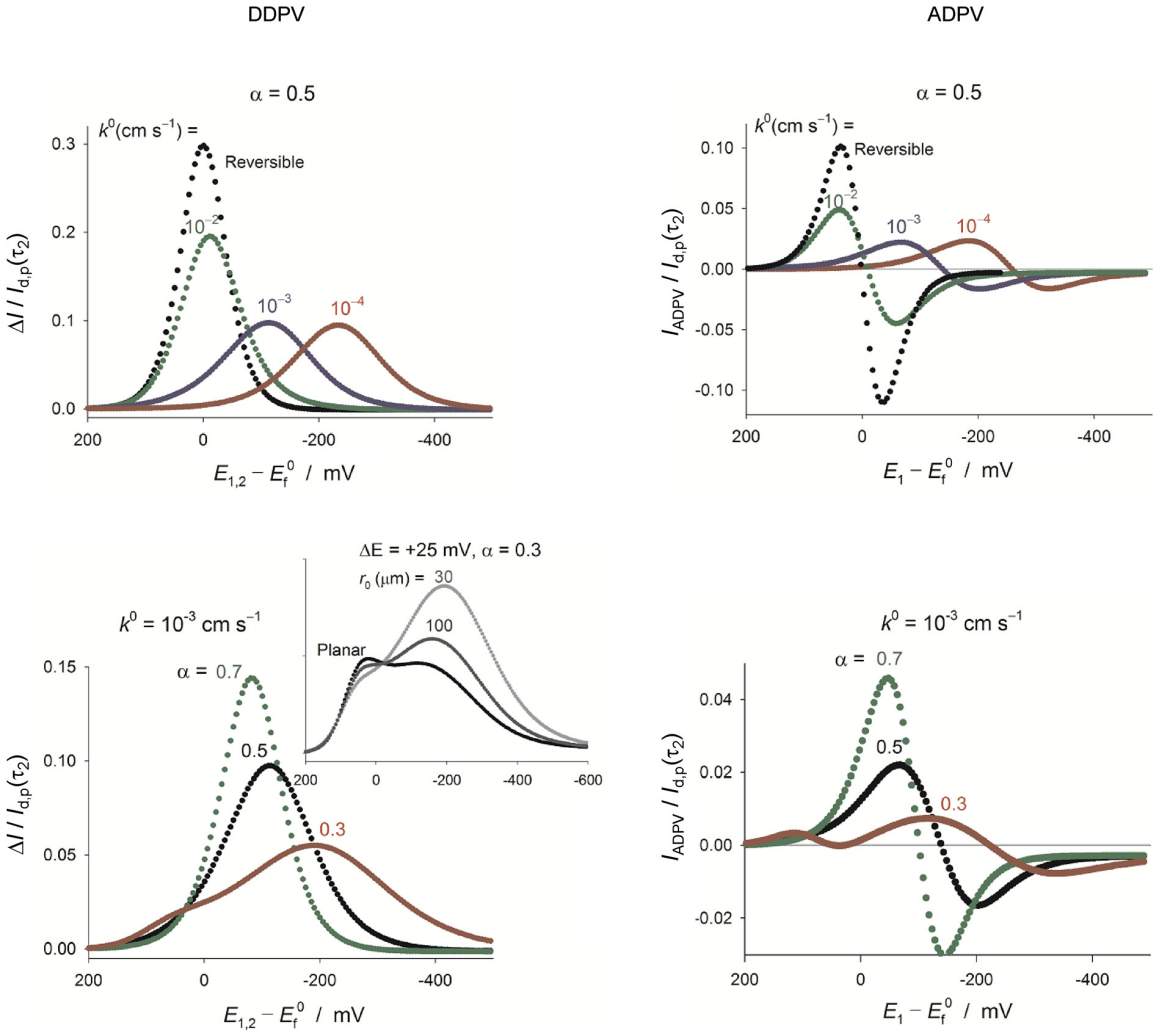
Influence of the Butler–Volmer kinetic parameters on the response of a one-electron reduction reaction in DDPV (left) and ADPV (right) at a (hemi)spherical microelectrode (*r*_0_=30 μm). *t*_1_=1 s, *t*_1_/*t*_2_=50, Δ*E*=50 mV. *E*_1,2_=(*E*_1_+*E*_2_)/2, *I*_d,p_(*t*_2_)=*FAc*_A_* 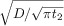.

The subtractive nature of the DDPV and ADPV techniques (introduced in the work of Molina et al.^84^) make them very valuable for quantitative analysis since the influence of background currents is further reduced, and peak-shaped responses are obtained. The influence of the electrode kinetics on the DDPV and ADPV signals are shown in Figure 6. The single-peak DDPV response and the double-peak ADPV signal shift to higher overpotentials, and the peaks become smaller and broader (larger half-peak width) as the electrode reaction transitions between the fully-reversible and the fully-irreversible limits. In the latter, the shape of the DDPV and ADPV signals is independent of the *k*^0^-value whereas the position does depend on *k*^0^. Regarding the influence of the transfer coefficient (*α*), the peak width increases, the peak height decreases, and the peak potential takes more negative values as the *α*-value is smaller in the case of electroreduction processes. The splitting of the DDPV and ADPV curves of electro-reductions with *k*^0^-values within the range 10^−3^–10^−4^ cm s^−1^ and very small transfer coefficients (*α*<0.3, see Figure 6) predicted at macroelectrodes^85^ have also been found at microelectrodes,77c though it gradually disappears as the electrode size is reduced. Note that in the case of electro-oxidation processes the splitting is predicted for large *α*-values (*α*>0.7). It is also worth mentioning that the splitting is not predicted by the symmetric Marcus–Hush kinetic model.70a

In practice, deviations from the fully-reversible behavior can be detected by comparison of the experimental results with those predicted for fast electron transfers with equal diffusion coefficients for the reduced and oxidised species:

DDPV

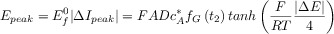
22

ADPV

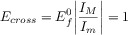
23

with *f*_G_(*t*_2_) being a time function, the form of which depends on the electrode geometry.^86^ Note that in Equation 20, it is considered that the arithmetic average (*E*_1_+*E*_2_)/2 is chosen for the *x*-axis potential.^87^

With respect to the peak currents, the magnitude of the peaks obtained with positive (Δ*E*>0) and negative (Δ*E*<0) pulse amplitude in DDPV are the same for reversible systems, as well as the heights of the maximum (*I*_M_) and minimum (*I*_M_) in the ADPV signal ([Eqs. (20), (21)] and Figure 6).^88^ These behaviours do not hold for finite-kinetic electrode reactions.^88^ Note that this reversibility criterion may not be conclusive for systems where the diffusivities of the oxidised and reduced species differ significantly. In such cases, even if the electron transfer is reversible, the position of the DDPV and ADPV signals depends on the double pulse duration and the electrode size and the values of |Δ*I*_peak_(Δ*E*<0)/Δ*I*_peak_(Δ*E*>0)| and |*I*_M_/*I*_m_| differ from 1.

The quantification of the electrode kinetics is possible from single-point fitting of the DDPV and ADPV curves.77a–77c For this, the peak height (in DDPV) and maximum current (in ADPV) are more sensitive in the case of quasi-reversible processes whereas the DDPV peak potential and the ADPV crossing potential are more appropriate for irreversible electrode reactions. Thus, from the fitting of the variation of the DDPV peak current and potential or the ADPV maximum current and crossing potential with the duration of the double pulse (*t*_1_+*t*_2_), the kinetics of three electro-reduction processes of different reversibility were successfully determined using mercury micro-hemispheres as working electrodes: 3-nitrophenolate anion in DMSO, 3-nitrophthalate di-anion in DMSO, and europium(III) in H_2_O.77b The analysis of the “first pulse” and “second pulse” components of the DDPV curve has also been recently proposed for the investigation of the electrode kinetics.^89^

It is worth noting that the influence of the kinetic parameters on the response in differential multi pulse voltammetry (DMPV) is qualitatively analogous to that discussed above for DDPV, and that the fitting methodology proposed is also applicable. Nevertheless, quantitative kinetic analysis of the DMPV curves requires for the use of numerical simulation methods.^90^ Thus, although the DMPV method is generally preferred to DDPV given that equilibrium conditions are not recovered after each pair of pulses and so the time of experiments is shorter, the theoretical modeling and analysis of results are more complex due to accumulative effects. Only for reversible electrode processes or at ultramicroelectrodes are analytical solutions available for DMPV.^91^ In the case of nonreversible processes at planar electrodes or conventional microelectrodes, the superposition principle is not applicable due to the time-dependence of the surface concentrations, and numerical methods must be employed to simulate and fit the DMPV signal.3a

#### 3.1.2 Reverse pulse voltammetry (RPV)^92^

**Figure 7:**
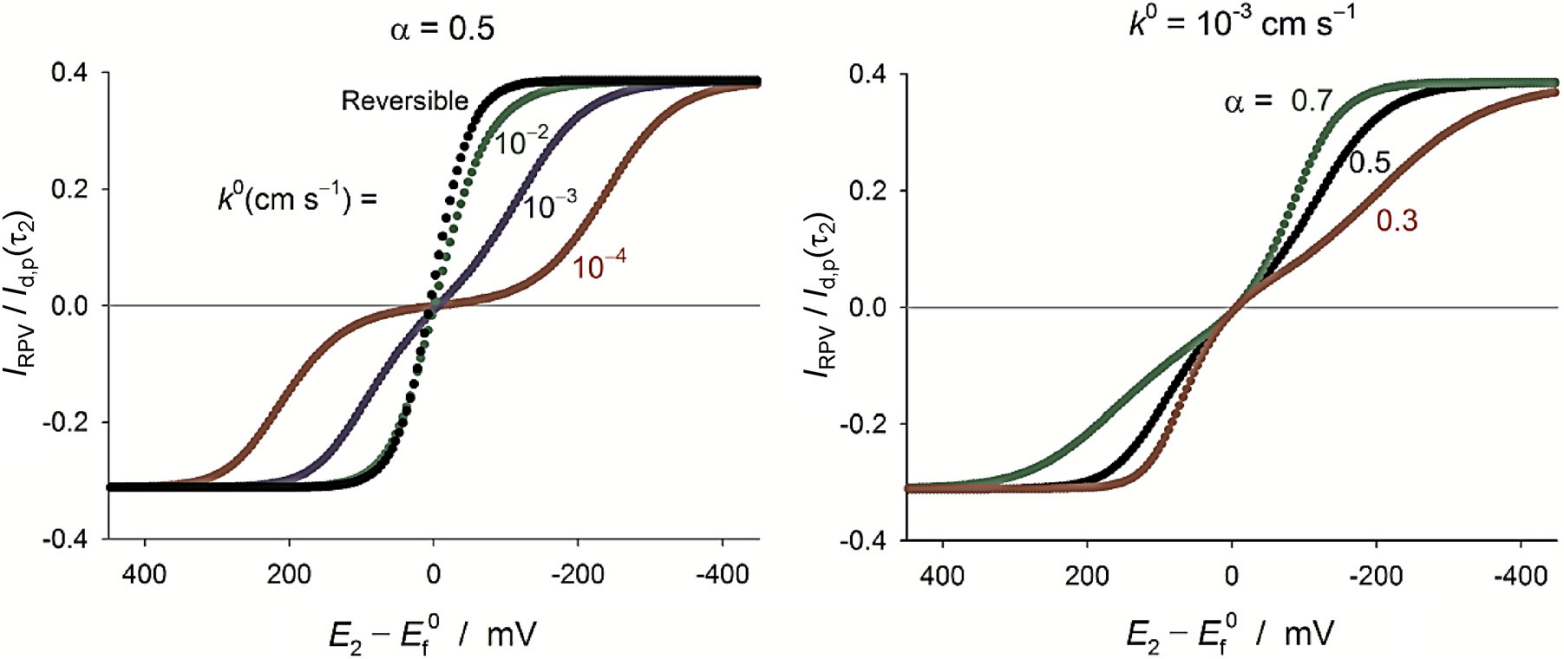
Influence of the Butler–Volmer kinetic parameters on the response of a one-electron reduction process in RPV at a (hemi)spherical microelectrode (*r*_0_=30 μm). *t*_1_=1 s, *t*_1_/*t*_2_=10. *I*_d,p_(*t*_2_)=FA 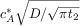.

The RPV response under transient conditions shows a cathodic and an anodic branch (without requiring the initial presence of the product species), and the shape of the RPV curve is greatly affected by the electron transfer kinetics as shown in Figure 7. As *k*^0^ decreases, the RPV voltammogram gradually splits into a cathodic and an anodic wave. Also, when the process is sluggish (*k*^0^<10^−3^ cm s^−1^) and the second potential pulse is long enough (*t*_2_≈*t*_1_), a maximum (“bump”) is observed in the anodic wave that is more apparent at large electrodes.77d With regard to the transfer coefficient, the *α*-value affects both the position and slope of the cathodic and anodic branches such that the cathodic wave is steeper and shifts to smaller overpotentials as *α* takes larger values (Figure 7), the opposite being true for the anodic wave. Therefore, visual inspection of the RPV curve enables us to estimate the electrochemical reversibility of the system, as well as the transfer coefficient. A summary of the reversibility criteria for DDPV, ADPV, and RPV is found in Table 1.

**Table 1:** Reversibility criteria for the DDPV,^88^ ADPV, and RPV techniques for electro-reduction processes when the diffusion coefficients of the electroactive species are equal. Note that in DDPV:
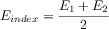
. The form of the functions *f*_G_(*t*) depends on the electrode geometry.^86^ *I*_d,p_(*t*_2_)=*F A* 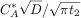.

	Fully reversible	Quasi-reversible	Fully irreversible
DDPV	• 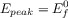	• 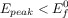	• 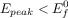
	• 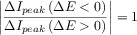	• 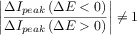	• 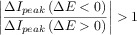
	• 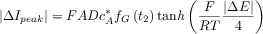	• 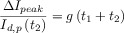 at macroelectrodes for a given *t*_1_/*t*_2_ value	• 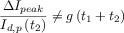 at macroelectrodes for a given *t*_1_/*t*_2_ value
			
ADPV	• 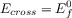	• 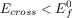	• 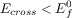
	• 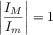	• 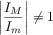	• 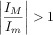
	• 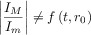	• 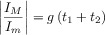 at macroelectrodes for a given *t*_1_/*t*_2_ value	• 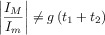 at macroelectrodes for a given *t*_1_/*t*_2_ value
			
RPV	• One wave	• Transition between one and two waves	• Two waves
			• Bump when *t*_2_≈*t*_1_

Analogously to the cases of DDPV and ADPV, it is possible to quantify the electrode kinetics parameters in RPV from the fitting of “singular” points of the curve. Thus, the values of the mid-wave potentials of the cathodic and anodic branches together with their variation with the double pulse duration have been employed with success for the kinetic study of the electroreduction processes mentioned in Section 3.1.1.

It is also worth noting that the cathodic and anodic limiting currents of the RPV curves are not affected by the electrode kinetics, and so they allow for simultaneous determination of the diffusion coefficients of both electroactive species. For this, an electrode of appropriate size must be employed (in the case of spherical electrodes: 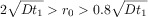), not being possible either at macro- or at ultramicro-electrodes unless the two electroactive species are initially present.^93^ Therefore, the use of microelectrodes of medium size in combination with the RPV technique enables the determination of the diffusion coefficients and the study of the electrode kinetics in a single experiment.

### 3.2 Square wave voltammetry (SWV)

Square wave voltammetry is well-known for its high sensitivity in electroanalysis, and it is also a powerful technique in the study of electrode kinetics and reaction mechanisms of solution-phase and surface-confined redox systems.^94^ SWV includes the benefits of differential pulse techniques along with those of potential sweep methods (fast experiments). The effects of the BV kinetic parameters on the SWV peaks are similar94a-95 to those described in Section 3.1.1 for DDPV such that the peaks are smaller, broader, and situated at larger overpotentials as *k*^0^ decreases and α decreases (in the case of electro-reductions) or α increases (for electro-oxidations).

Simple and rapid diagnosis criteria for the detection of finite electrode kinetics can be established based on deviations from the SWV signal expected for fully-reversible processes. Thus, the value of the peak current of nonreversible processes will be smaller than that predicted by the following expressions for reversible electrode reactions at disc, (hemi)spherical, band and cylindrical electrodes and microelectrodes under typical SWV conditions (E_SW_=50 mV, E_s_=5 mV):86b

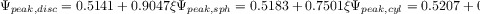
24

where 
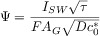
and 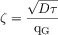, with τ being half the square wave period (τ=1/2 *f*) and *q*_G_ the characteristic dimension of the electrode: the radius for discs, spheres and cylinders and the half width for bands.

The value of the half-peak width (W_1/2_) is also a parameter of interest given that the W_1/2_-value for one-electron reversible processes is defined only by the SW amplitude (E_SW_, [Eq. (23)], W_1/2_ being independent of the electrode geometry and frequency employed:86b

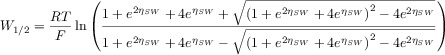
25

where 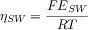
is the dimensionless SW amplitude. Thus, in absence of ohmic drop effects,^96^ experimental W_1/2_-values larger than those predicted by [Eq. (23)] indicate a nonreversible behaviour. The effect of the step potential (E_s_) on the SWV signal also offers a simple criterion to estimate the degree of reversibility.^97^ Thus, whereas the SWV response of reversible systems is scarcely affected by E_s_, the SWV response of irreversible reactions varies significantly with E_s_: the smaller the E_s_ value, the smaller the SWV peak.

**Figure 8:**
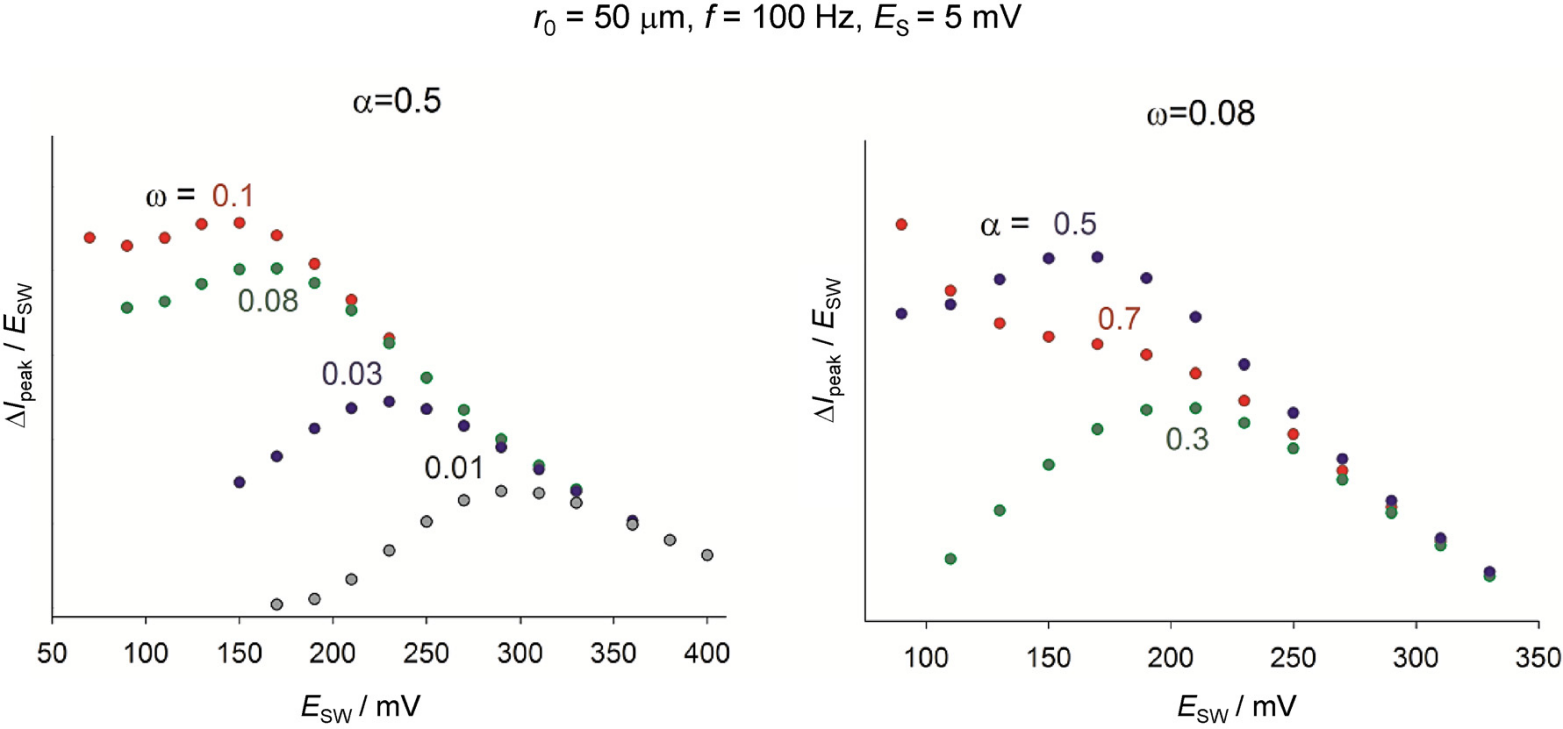
Influence of the electrode kinetic parameters on the amplitude-based quasi-reversible maximum of a one-electron reduction at a hemispherical microelectrode corresponding to a solution-phase redox system. 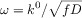.

For quantitative analysis of the SWV response of nonreversible electrode processes, numerical simulation methods are necessary,3a semi-analytical solutions in the form of a system of recursive formulae being also available.94a Though frequency-based approaches have been usually considered for the investigation of the electrode kinetics,94a Mircevski et al.94i have recently proposed a new approach based on the variation of the SW amplitude rather than the time scale of the scans. According to the new amplitude-based strategy, the electrode kinetics is characterised from the variation of the separation of the peak potentials of the forward and backward components of the potential-corrected SW voltammogram and/or the peak current of the net response with the SW amplitude (E_SW_). This variation is sensitive to the kinetic parameters as shown in Figure 8 for the amplitude-normalised peak current (ΔI_peak_/E_SW_). The amplitude-based methodologies have been applied with satisfactory results to the study of solution-phase and surface-confined3a,^98^redox systems.

**Figure 9:**
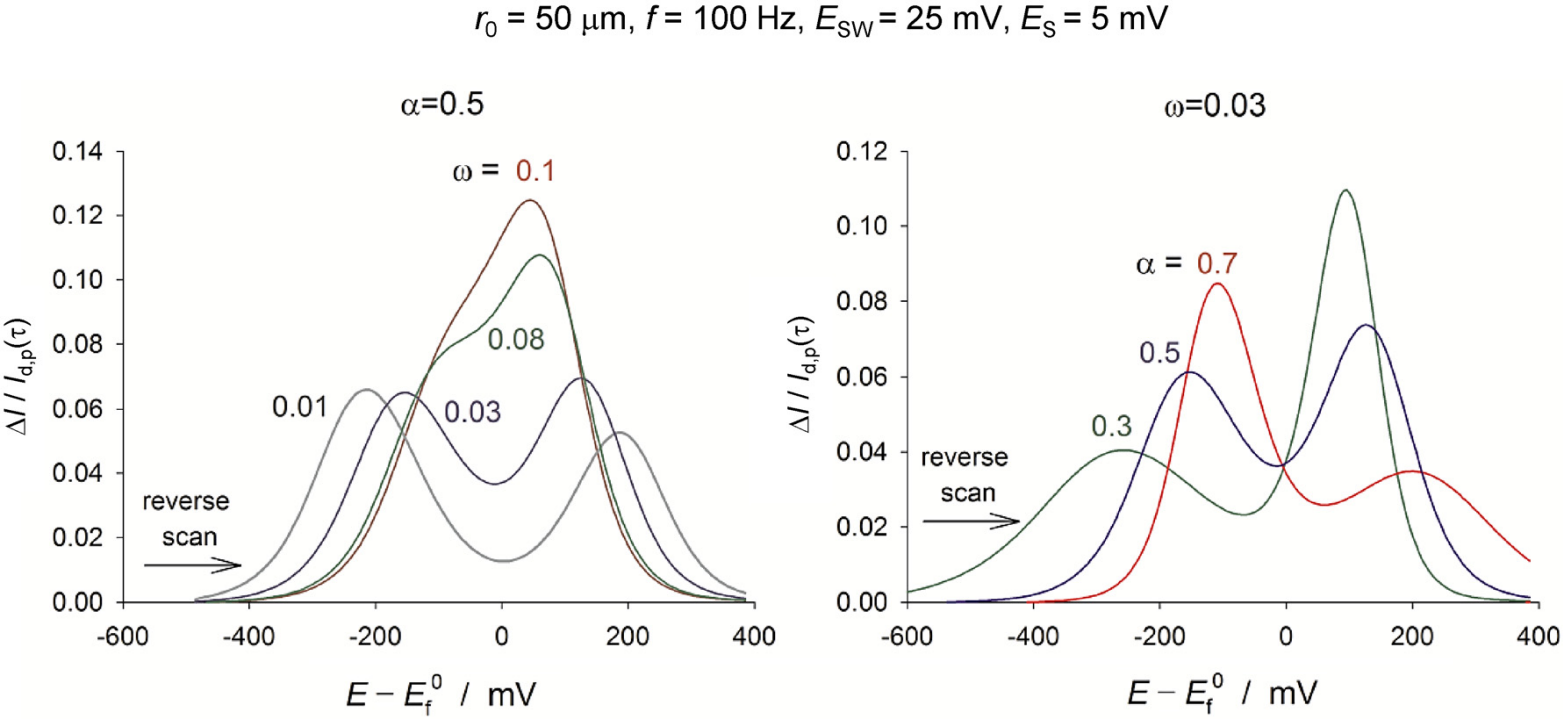
Influence of the electrode kinetic parameters on the reverse scan of the cyclic SWV response of a one-electron reduction corresponding to a solution-phase redox system. 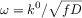, 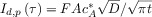.

Cyclic and reverse scan SWV has also been proposed and employed in quantitative kinetic studies.^69^,^71^,^99^ In the case of quasi-reversible and irreversible diffusional processes (Figure 9), a double peak is observed in the reverse scan at negative (cathodic nature) and positive (anodic nature) potentials with respect to the formal potential. The splitting of the peak is more apparent as the electrode size and/or the frequency increase and they separate as the electron transfer is more sluggish (Figure 9). The fitting of the peak potentials, peak widths, and relative peak height enables the characterisation of the electrode kinetics. This approach has been applied to the study of the electroreduction of 2-nitropropane^71^ and europium (III)99a on mercury electrodes and microelectrodes.

### 3.3 Cyclic pulse voltammetries

Software packages of modern electrochemical instrumentation enable the researcher to “customize” the voltammetric perturbation applied to the system. Within this context, Jadresko et al.^100^ have recently proposed two new variants of pulse techniques: cyclic multi pulse voltammetry (CMPV) and cyclic differential multi pulse voltammetry (CDMPV). The potential-time program is analogous to that employed in normal/reverse pulse voltammetry and DDPV, respectively, with the key differences that the perturbation is applied in a “cyclic mode”, and that equilibrium conditions are only restored at the end of the experiment. As a result, the characterisation of the system is more complete and sound, and the electrochemical measurements are faster. The resulting signal enables qualitative analysis of the process (including the electrode kinetics) from visual inspection of the voltammograms as well as quantitative analysis from single-point fittings.^100^

### 3.4 Staircase versus analogue cyclic voltammetry

Since the early 90s most commercial potentiostats have been predominantly digital, computer controlled devices. In part due to cost and ease of implementation the basic cyclic voltammetric technique provided by these devices involves the application of a ‘staircase’ ramping potential. The use of a staircase waveform for voltammetry was initially proposed as a route by which Faradaic and capacitive currents may be more readily experimentally discriminated between (cf. voltammetric pulse techniques).^101^ This discrimination is partially enabled on the basis of the differing time constants associated with diffusional redox (*t*^−0.5^, for a linear mass-transport regime) and capacitive charging (e^−t^, in the heavily simplified RC circuit analogy) processes. However, prima facie there is no reason to assume that staircase and analogue cyclic voltammetry are equivalent.

**Figure 10:**
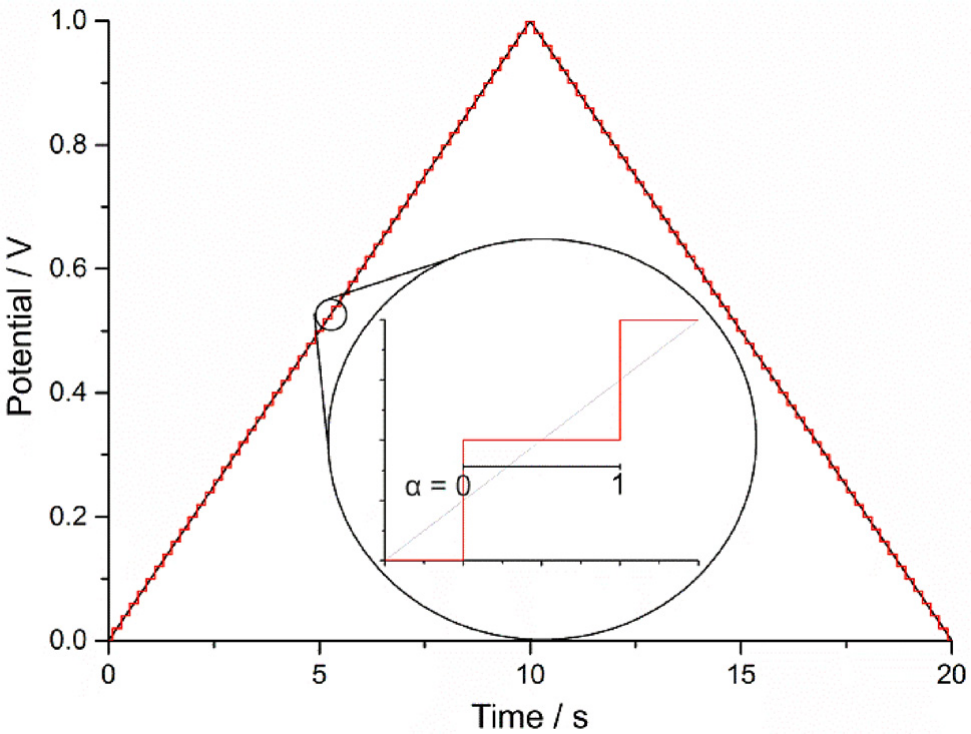
Comparison of the voltage wave forms used for staircase (red) and true analogue (black) cyclic voltammetry. Zoomed inlay depicts an individual step showing the sampling alpha scale. When *α*=1 the current is sampled at the end of the step; alternatively, *α*=0 implies a current measurement at the beginning of the step. Data depicts the wave form used for a cyclic voltammogram (0–1 V) at a scan rate of 0.1 V s^−1^ and with a step potential of 20 mV.

Figure 10 depicts the variation of the potential used for ‘staircase’ (red) and true analogue (black) cyclic voltammetry. For a given electrochemical system studied via analogue cyclic voltammetry (CV), the measured response is simply a function of the scan rate (assuming appropriately chosen start, finish, and turning potentials). Conversely, for staircase cyclic voltammetry (SCV), the resulting voltammogram is a function of the scan rate (step height/step time=V s^−1^), the step size (*E*_step_/V), and the point (or points) at which the current is sampled during each step. In the case that the current is sampled once during each step the time at which the current is sampled is expressed as the dimensionless value alpha (*α*), where an alpha value of one or zero implies the current is sampled at the end or beginning of each step respectively (see inlay of Figure 10). This sampling alpha value bears no relation to the transfer coefficient and the two should in no way be conflated!

In the late 80s, Osteryoung published a series of papers investigating the differences and possible equivalences between staircase and analogue cyclic voltammetry.^102^ Importantly, for diffusional redox species the use of SCV tends to lead to voltammetric waves that exhibit larger peak-to-peak separations and suppressed peak heights. It should be recognised that all analytical expressions (the Randles–Ševčík equation for example) and commercially available simulation packages assume the utilisation of an analogue potential ramp. Consequently, the use of these equations or simulation software for quantification of SCV results can lead to erroneous results.

For a reversible diffusional process, equivalency (within experimental error; peak current, *I*_p_ error <3 %, peak position within 2 mV) between SCV using an alpha value of 1 and analogue CV reportedly requires the use of a step size of 0.26 mV.102a However, for diffusional processes this constraint may be relaxed through the use of an alpha value of 0.25–0.3,^103^ enabling the use of slightly larger step potentials without too significant a deviation from the results predicted for analogue CV. Depending on the experimental conditions and the equipment used, it may or may not be possible to select experimental parameters that allow the SCV response to closely approximate that obtained from analog CV. Although the response is improved by using an alpha of 0.3, for situations in which accuracy is highly pertinent it may be advisable to revert to using true analogue cyclic voltammetry;^104^ alternatively one may explicitly simulate the response accounting for the staircase ramping potential.^105^ Recent theoretical studies have investigated the influence of the alpha value in staircase voltammetry for the case in which the diffusion profile at an electrode is transitional between the linear and convergent limits.^105^ Moreover, expressions for the analytical solution at a microelectrode for the staircase response of single-, multi-, and catalytic electron transfer processes have also been provided.^81^,86a,^106^

**Figure 11:**
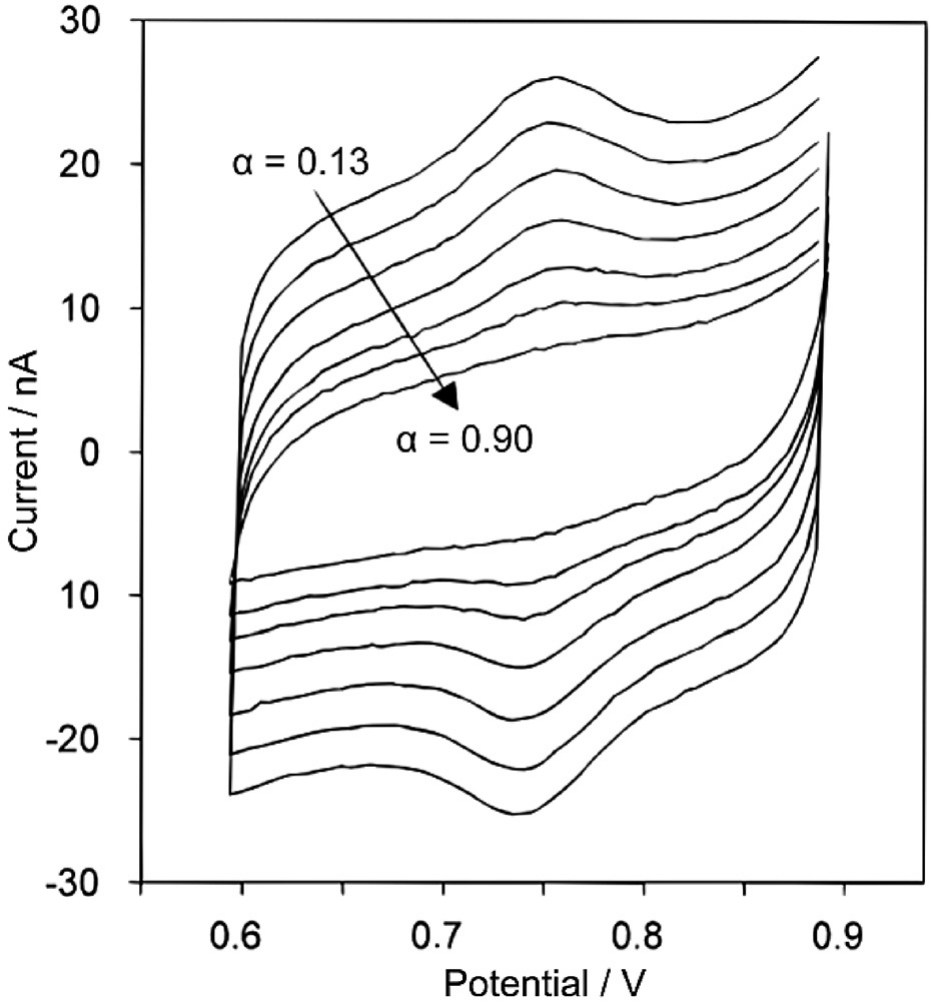
Staircase voltammograms of yeast cytochrome c peroxidase (CcP) at different sampling times. Conditions: 0.13 μm CcP in 20 mm sodium acetate, pH 5.45, 0 °C, *ν*=20 mV s^−1^, *E*_step_=5.04 mV, and *α*=0.13, 0.15, 0.18, 0.25, 0.35, 0.48, and 0.90. Each trace is the average of four cycles. Adapted with permission from Ref. 107. Copyright 1999, American Chemical Society.

The above discussion has focused on the voltammetric response of *diffusional* redox processes, where for many systems the application of SCV yields qualitative but *not* quantitative correspondence with the analogue technique. In contrast, for surface-bound species, the obtained voltammetric results can differ profoundly between the techniques. In the most extreme cases, where the surface species exhibits fast electron transfer kinetics, it may arise that the Faradaic charge transfer occurs prior to the measurement point on the step. In this situation the use of SCV may yield a voltammogram that is completely devoid of a voltammetric feature even if the redox species is present. To exemplify this point, Figure 11 shows the staircase voltammetric response of cytochrome c peroxidase on a pyrolytic graphite electrode, where the experimental sampling alpha value has been varied between 0.13–0.9.^107^

For situations in which the current is sampled towards the end of a step, the reversible cyctochrome c peroxidase voltammetric wave is not recorded. SCV of a surface-bound feature only becomes equivalent to analogue CV when the scan rate (*v*) is greater than 10 *k*^0^*E*_step_.^107^ Subsequently, the use of staircase voltammetry for the investigation and quantification (in terms of surface coverage) of a reversible surface bound process must be approached with caution. To this end it is noted that one of the prime examples of such a system ‘misrepresented’ by SCV is encountered with hydrogen UPD on platinum,^108^ however other molecular species can encounter similar problems.^109^ This is particularly true when investigating surface-bound species as a function of temperature, where it may be found that at higher temperatures the surface-bound redox-wave is essentially ‘lost’ when using SCV. This can occur simply due to the increase in the electron transfer kinetics as a function of temperature. One method by which these problems may be circumvented is by sampling the current continuously over the course of a potential step and averaging the result; for surface bound species this results in a voltammogram closely comparable to that found with the use of analogue CV. Depending on the potentiostat manufacturer, this technique goes by a variety of names including ‘current integration’ and ‘surface mode sampling′. Finally, when investigating the fundamentals of the electron transfer process of surface-bound species by voltammetric techniques, if a staircase potential ramp has been used, it is imperative that this is taken into account in the simulations so as to ensure validity of the results.^110^

### 3.5 Insights into the concept of the diffusion layer thickness

The Nernst diffusion layer concept (or linear diffusion layer) provides a useful approach to the species concentration profiles and the diffusive mass transport in electrochemical systems. The thickness of such a layer, *δ*, informs about the extent of the region in solution where concentration changes take place and the efficiency of diffusion under given experimental conditions. This information is essential in digital simulation of electrochemical experiments,3a the evaluation of possible interferences due to convective mass transport^111^ and double layer effects,^112^ and the design of micro- and nanoelectrode arrays in order to predict the overlapping between adjacent diffusion domains.^113^ Nevertheless, only very recently have the effects of finite electrode kinetics and convergent diffusion on *δ* been investigated.^114^ Thus, analytical expressions have been reported for the study of the linear diffusion layer thickness in any voltammetric experiment.

#### 3.5.1 Nonplanar diffusion in any voltammetric technique

**Figure 12:**
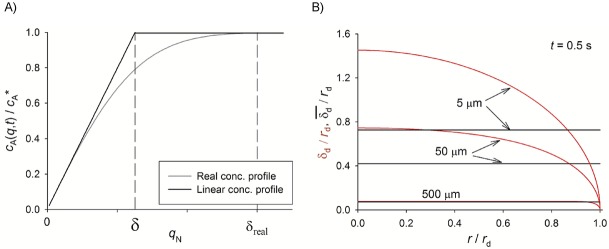
A) Schematic of the linear and real diffusion layer thicknesses in uniformly accessible electrodes. B) Dimensionless linear diffusion layer thickness (red line) and dimensionless *average* linear diffusion layer thickness (black line) at disc electrodes.

In the case of uniformly accessible electrodes, such as (hemi)spheres and cylinders, the diffusion problem can be reduced to a single spatial coordinate corresponding to the direction normal to the electrode surface, *q* (Figure 12). The linear diffusion layer thickness is defined as the distance to the electrode surface where the linear concentration profile takes the bulk value c* (Figure 12 a). Accordingly, the *δ* value is given by:

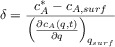
26

Taking into account that for a fully reversible electron transfer A+*e*^−^⇌ B, with species A and B having equal diffusion coefficients, the surface concentrations only depends on the applied potential, *E*_p_, such that when *c*_B_*=0 it is fulfilled that:

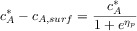
27

where 
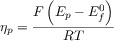
, and that the surface concentration gradient can be expressed as follows after a sequence of *p* potential pulses:

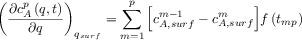
28

where 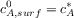, *t*_mp_=(*p*−*m*+1)τ and *f*(*t*_mp_) is a time function, the form of which depends on the shape of the electrode employed.^86^

From Equations 24–26, the following expressions are obtained for the linear diffusion layer thickness at planar, (hemi)spherical, and cylindrical electrodes in any voltammetric technique consisting of a sequence of *p* potential pulses of the same duration, *τ*:

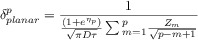
29

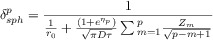
30

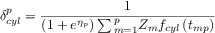
31

where:

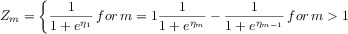
32

At microdiscs and microbands, the mass transport of species in solution towards/from the electrode is not uniform over the whole electrode area. Thus, the mass flux is higher at the electrode edge than at the electrode centre, and the linear diffusion layer thickness has an average character. After the application of a sequence of *p* potential pulses of duration *τ*, the average linear diffusion layer thickness for a reversible process is given by:

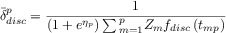
33

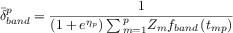
34

**Figure 13:**
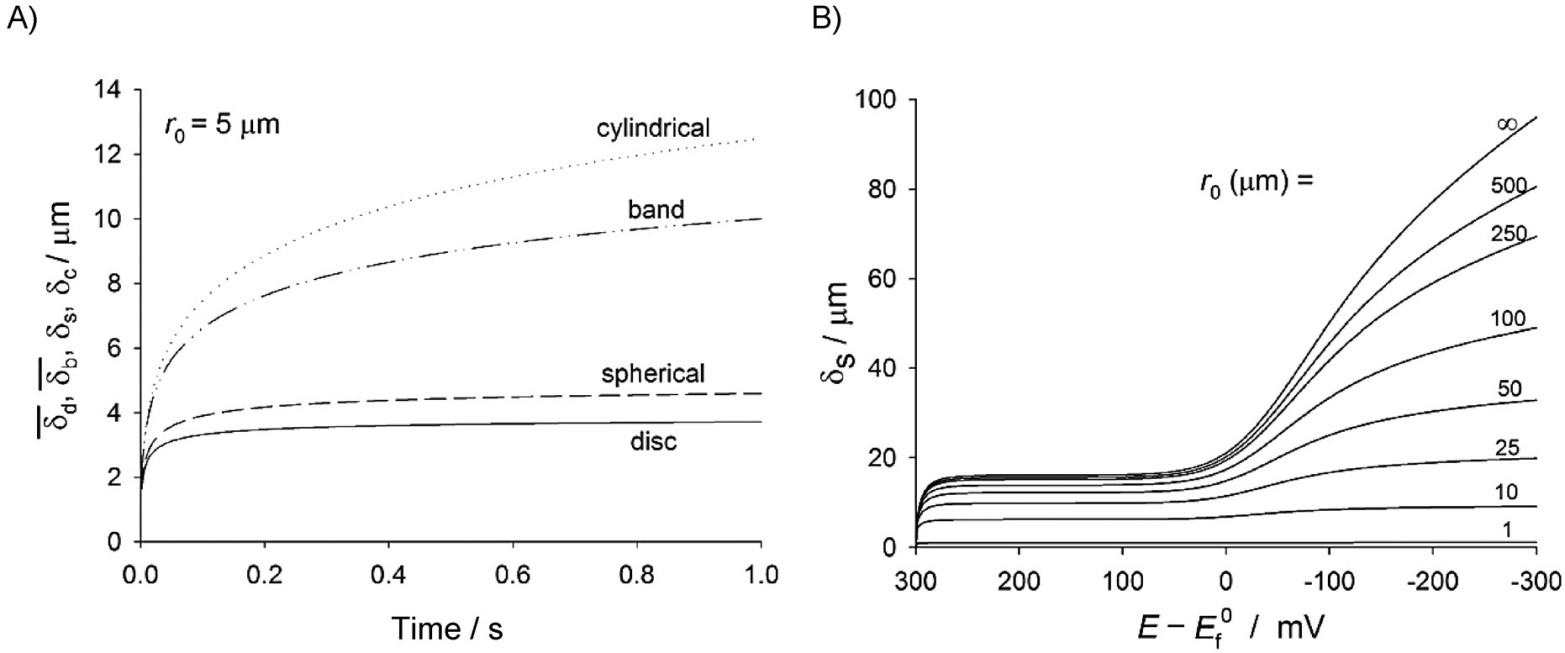
Evolution of the (average) linear diffusion layer thickness in A) single-step chronoamperometry at different microelectrode shapes and B) linear sweep voltammetry at spherical electrodes of different radii at 100 mV s^−1^.

Equations 27–29 and 31–32 enable the study of the behaviour of the linear diffusion layer for very different electrodes geometries and voltammetric techniques. Figure 3 shows the evolution of the (average) linear diffusion layer thickness in chronoamperommetric (Figure 3 a) and linear sweep voltammetry (Figure 3 b) experiments at microelectrodes of different shape (Figure 3 a ) and different radii (Figure 3 b). In both experiments, the thickness of the linear diffusion layer increases as the experiment proceeds and so the duration of the perturbation. Regarding the electrode shape (Figure 13 a), for a given *r*_0_, the *δ* values coincide for any geometry at very short times when diffusion is predominantly planar, with differences between them becoming more apparent with time. Thus, *δ* decreases in the order: cylindrical>band>spherical>disc, which means that the mass-transport efficiency (current density) follows the inverse order. With respect to the influence of the electrode size (Figure 13 b), the thickness of the linear diffusion layer in absolute terms decreases as the electrode shrinks, though the thickness relative to the electrode radius (i.e. δ/*r*_0_) increases, and it tends to 1 at microelectrodes.

It is also important to mention that *δ* must be taken cautiously as an estimation of the *real* diffusion layer thickness given that these two magnitudes diverge very significantly at microelectrodes and nanoelectrodes. Thus, whereas the ratio *δ*_real_/*δ* is found to be about 2 at macroelectrodes (planar diffusion), it is about 15 at conventional microelectrodes (a few micrometer-radius), and it tends to 100 at ultramicroelectrodes (steady-state conditions).114b

#### 3.5.2 Finite electron transfer kinetics

For the evaluation of the impact of the electrode kinetics on the linear diffusion layer thickness, the following expression has been deduced for processes of any degree of reversibility in single potential-step chronoamperometry at (hemi)spherical electrodes of any size:114b

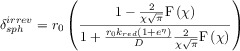
35

where:

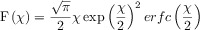
36

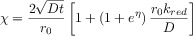
37

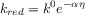
38

The degree of reversibility has a profound influence on the species surface concentrations, which are time-dependent for nonreversible processes (unlike for fast electron transfers, [Eq. (39)]):

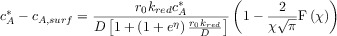
39

as well as on the behaviour of the linear diffusion layer thickness, which is potential-dependent as can be inferred from Equations 33–36.

The results obtained from Equation 33 shows that the linear-diffusion layer thickness of nonreversible processes is smaller than for reversible electron transfers (
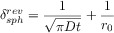
), except under limiting current conditions and at ultramicroelectrodes (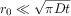) where *δ*_sph_=*r*_0_ for any electrode kinetics, applied potential, and electrochemical method. In any other situation, 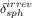
is smaller than 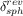
and it varies in the range: 
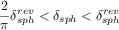
.

## Section 4: Diffusion and Random Walks

### 4.1 Modelling diffusional processes

When it comes to the experimental validation of reaction mechanisms (such as the mechanisms discussed in Sections 1 and 2), digital simulations are a frequently used tool in todays electrochemical and electroanalytical research. Since simulations can be specifically designed to predict experimental data of a certain electrochemical system based on a number of different models of underlying fundamental processes, they may provide data for direct comparison with experimentally-obtained results. Herein, employed simulations always combine two models: a model for charge-transfer processes at solid–liquid boundaries and a model for the mass transport of the analyte. Such interface processes may, for instance, include electrochemical interactions according to kinetic models such as the above-discussed Butler–Volmer or Marcus–Hush models, or other physiochemical processes like adsorption and desorption kinetics, which provide the boundary conditions for the mass-transport problem. In the common case that convective processes are negligible, mass transport can be modelled through Ficks second law:^115^

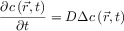
40

where *c* is the concentration and *D* the isotropic diffusion coefficient of the analyte. This equation can be solved via a number of different methods, most prominently through finite differences3a or finite elements,3c which both provide solutions for the concentration profile 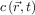. A so-obtained solution for the concentration profiles of reacting species then allows the calculation of the expected average current across all electrochemical interfaces from the concentration gradient at the respective interface, which is the desired result in most applications. However, the systems intrinsic noise characteristics cannot be directly modelled through finite-difference or finite-element approaches as such noise characteristics are due to the discrete nature of the analyte, which results in a stochastic charge transfer across the interface, being particularly relevant at low concentrations or small structure sizes.

Concentration profiles can rather be interpreted as probability densities of finding a particle at a certain position, but do not allow direct insights into the stochastic nature of the charge-transfer process at the interface. One way to overcome this issue is the use of the random walk method. In this approach, the pathways, as well as all electrochemical interactions, of each analyte molecule are modelled individually. Initially at *t*=0, all analyte molecule *i* positions 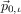
are set by randomly distributing molecules according to the initial concentration profile within the simulated space. The concentration profile can then be written in the form:

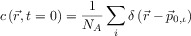
41

where 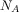
represents the Avogadro constant and 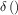
the Dirac delta function. For *t*>0 the Dirac delta function, which describes the exact initial positions, can be replaced by the Greens function *G* of the linear differential operator 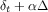
with α ∈ ℝ, which corresponds to the differential operator in the diffusion equation. This Greens function is given by:

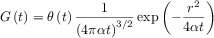
42

where 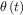
is the Heaviside step function. We then obtain a continuous concentration profile as a function of time:

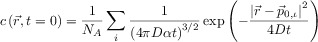
43

which again provides a probability density of the particles’ positions instead of desired discrete positions. However, in contrast to the direct solution of Ficks second law, this result describes the temporal evolution of the concentration profile with defined initial positions for each modelled molecule.

In order to transform this finding into an exact distribution of molecules, the average displacement 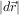
of a particle after a given time *dt* is calculated from the Greens function. Via the investigation of the mean squared displacement of an individual particle, we obtain:

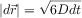
44

for the three-dimensional case. In the one-dimensional case, we calculate:

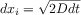
45

from the mean squared displacement.^116^

The temporal evolution of exact particle positions that fulfil the diffusion equation can hence be found by substituting each particles spatial probability density function by random displacement after discrete time steps of the width *dt*. Mathematically, this approach can be expressed as:

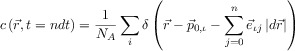
46

where 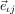
is a random unit vector. For reasons of computational simplicity, however, the distribution of particles in two- or three-dimensional systems are often expressed in terms of independent one-dimensional average displacements, 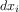, in each dimension. Based on this assumption, the previous equation is transformed to:

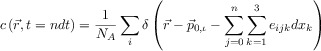
47

where 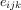
is a one-dimensional random unit vector featuring the values +1 or −1. Since this result provides exact stochastic positions of all active molecules at any time, the noise characteristics of the modelled system can be simulated in great detail and in addition to the expected average values that can be obtained from finite differences or finite elements.

While random walk simulations offer the advantage of the ability of noise modelling, which is not offered by many other methods, the random walk approach features two main disadvantages. First, since every molecules pathway has to be modelled individually, the computational effort scales with the number of active molecules in the modelled system. The random walk approach is hence not suitable to model high concentrations or large systems. Secondly, the appropriate definition of boundary conditions may be difficult as the above discussed theoretical justification of the random walk approach implies a significant limitation: The Greens function approach chosen in Equation 41 solely describes the temporal evolution of a diffusing particle in the absence of diffusion boundaries. In order to model a real electrochemical set-up including electrodes and inactive surfaces, approximations must be made.

**Figure 14:**
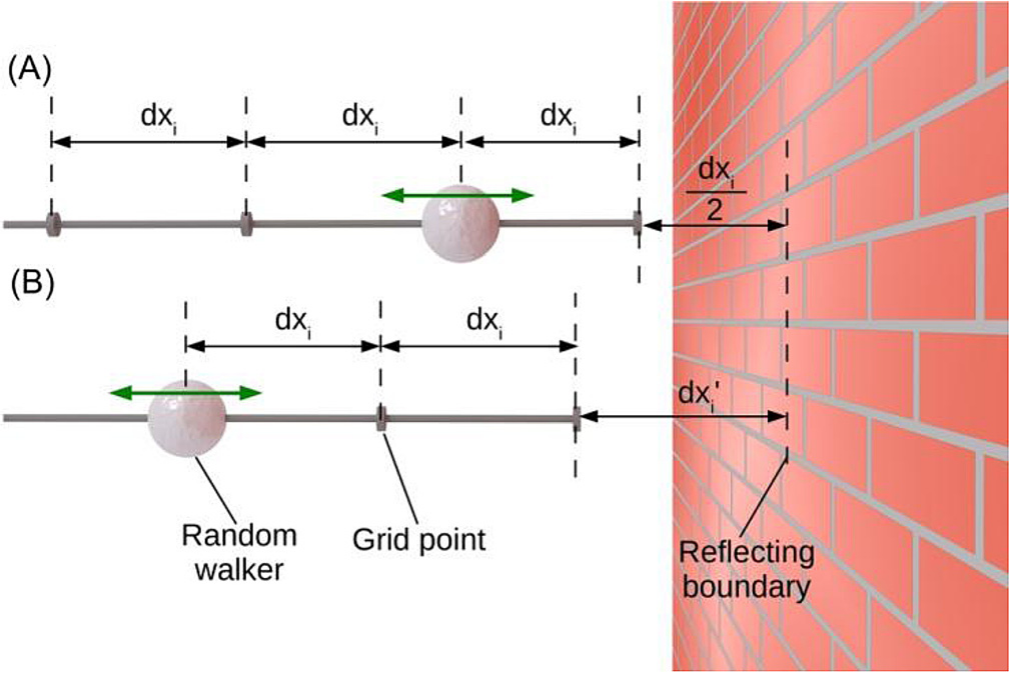
Examples of two different definitions of boundary conditions in a random walk simulation. The grid in (B) is displaced relative to the boundary.

To illustrate this problem, we focus on the common case of a random walker on a one-dimensional grid in between two reflecting boundaries; the approach is, however, equally applicable to three dimensions. In the one-dimensional case, the boundary condition at a boundary can be formulated in multiple ways; the most common definition can be seen in Figure 14 A). The closest grid point to the boundary is separated from it by 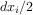
and, during each temporal step *dt*, the random walker may either remain on this position or perform a step away from the boundary. In this case, the reflection at the boundary has to be divided into two independent first passage problems: the diffusive movement to the boundary surface and the movement back to the initial position of this step. Mathematically, however, the expected time of such a reflection, *dt*_0_, does not equal the time, *dt*, as it is presumed in the formulation of the boundary condition. Using Equation 43, we obtain:

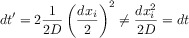
48

Such a boundary condition hence induces an error that scales with the spatiotemporal step width of the random walk. In order to reduce this error, the distance of boundary to the closest grid point of the random walk must then be corrected to *dx’* as it can be seen in Figure 14 B):

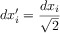
49

as was discussed by Kätelhön et al.^117^ If, however, computational effort is not a limiting factor of the simulation, the simplest way to circumvent the problem of the definition of appropriate boundary conditions to choose a sufficiently small spatial step width for the random walker. Since the induced error scales with the spatiotemporal step width of the simulation, the deviation from the analytical result will scale with *dt* and 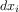. When simulating a large number of molecules or a long experiment, this leads to a significant increase in computational effort and is therefore often not applicable.

In recent years, the noise modelling capabilities of the random walk approach have been exploited in a number of different studies focusing on electrochemical systems. These studies include more fundamental analyses of stochastic versus statistic descriptions of diffusion processes^118^ as well as the description of experimental systems. Cutress et al. for instance used a GPU-based random walk simulation to investigate cyclic voltammetry^119^ and potential-step chronoamperometry^120^ at low concentrations, while Kätelhön and Compton focused on the noise-characteristics of the mediated Faradaic current across a nanoparticle impacting on a Faradaically inactive electrode surface.^121^ Aside from the modelling of systems at low concentrations, random walk simulations can further be employed to investigate noise characteristics of electrochemical sensors in nanofluidic devices. Hereby, applications include the modelling of methods for single molecule detection^122^ as well as the simulation^117^,^123^ of spectra obtained from electrochemical correlation spectroscopy (ECS).^124^

### 4.2 From molecules to nanoparticles

Aside from its application in modelling diffusion of molecular probes, the random walk approach can be used to simulate the Brownian movement of nanoparticles.^125^ Here, random walks are particularly helpful, since experiments focusing on the mass transport or electrochemistry of nanoparticles, such as nano-impacts, are usually performed at concentrations that are sufficiently low to resolve the reaction of individual nanoparticles. Currents recorded in such experiments are hence strongly influenced by the stochastic nature of the system, which can be modelled through random walks.

When modelling the diffusion of nanoparticles, the above discussed approach has to be slightly modified in order to account for the nanoparticles’ distinct diffusional characteristics that differ from molecular diffusion: Due to their greater size, nanoparticles are affected by the effect of near-wall hindered diffusion when they approach a diffusional boundary. This effect leads to an anisotropic diffusion coefficient distinguishing between diffusion perpendicular and in parallel to the boundary. The diffusion coefficient 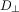
in the perpendicular case is then given by:

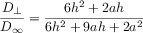
50

where *a* is the radius of the particle, *h* the particles elevation from the surface, and 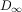
the bulk diffusion coefficient.^126^ The latter can be approximated well via the Stokes–Einstein equation:

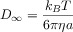
51

where 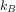
is the Boltzmann constant, *T* the temperature, and 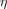
the viscosity of the solvent.^26^ The parallel component of the diffusion coefficient *D*_∥_ can be described through:^127^

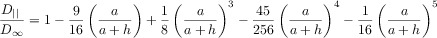
52

**Figure 15:**
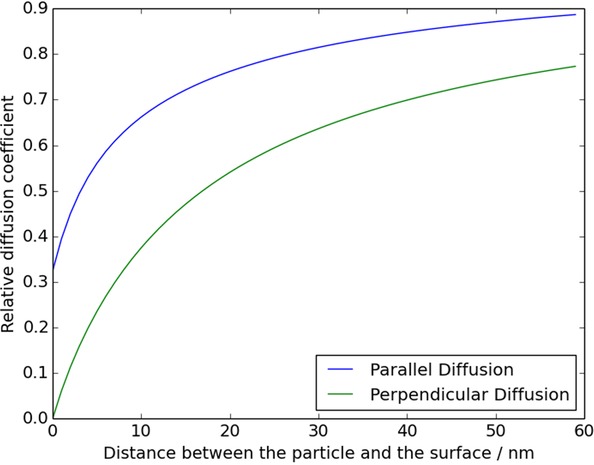
Diffusion coefficients perpendicular and in parallel to a boundary relative to the bulk diffusion coefficient, (*D*(*h*)_(⊥,||)/*D*_∞). Presented graphs were for a particle featuring a radius of 15 nm..

The distance-dependency of both diffusion coefficients can be found in Figure 15.

As it can be seen in the plot, perpendicular diffusion slows down near the boundary and eventually vanishes at the surface. Diffusing particles that are located in this area hence spend on average a significantly longer time in this zone than in any other zone of equal size in the bulk reservoir. This effect of hydrodynamic adsorption was recently discussed generally and with respect to the average time of residence that a catalytically active particle spends within the zone of electron transfer near an electrode.125b

## Section 5: Modelling Migration and Diffusion

The vast majority of electrochemical experiments are carried out in the presence of a large excess of inert, fully dissociated electrolyte.^78^ The purpose of this electrolyte is to generate a high ionic strength in solution, which will efficiently dissipate the excess charge necessarily introduced into solution via electrolysis, and suppress the resultant electric field. There are two main reasons that this is normally the case.

**Figure 16:**
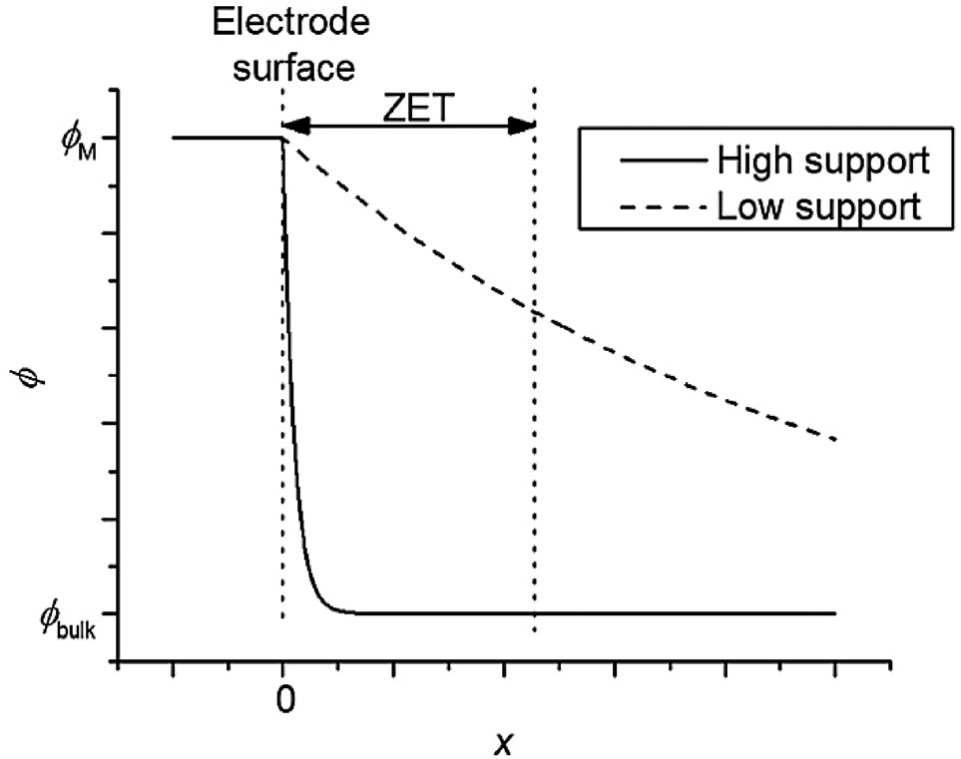
Schematic representation of solution potential profiles under conditions of high (solid line) and low (dashed line) support. The dotted lines represent the zone of electron transfer extending from the electrode surface out a certain distance into solution.

The first reason is to prevent ohmic drop.^26^ The driving force behind electron transfer is the potential difference between the electrode and the point in solution where electron transfer takes place, 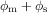. The bulk solution, far from the electrode, has some fixed potential 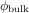. If the potential drop between 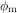
and 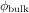
occurs over a distance greater than the electron tunneling distance for electron transfer (outside the zone of electron transfer, ZET) then the full driving force will not be felt; the potential difference is lowered as a result of ohmic drop. If a large amount of excess electrolyte is added to efficiently dissipate excess charge, the distance over which the drop between 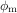
and 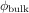
occurs is compressed to a distance much smaller than the ZET. This being the case, the electron transfer is then driven by the maximum potential difference, 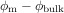. This is exemplified schematically in Figure 16.

The solid line shows the potential profile under conditions of high support (a large excess of supporting electrolyte), which is compressed to short distances and does not extend very far into the ZET. The dashed line shows low support conditions (small amounts of supporting electrolyte), where the electric field extends out beyond the ZET, resulting in a smaller driving force for electron transfer.

Secondly, a compressed electric field will eliminate migratory effects from the mass transport of solution phase species.^128^ If an electric field extends far beyond the electrode surface, the resulting potential gradient in solution will induce electrical migration of charged species, in addition to diffusion. The majority of analytical theory in electrochemistry assumes diffusion-only conditions, and the presence of migration complicates matters. For these two reasons, excess supporting electrolyte is usually added to an electrochemical experiment. However, migration effects can offer extra kinetic and mechanistic information unavailable at high support levels.^129^ For this reason, it may be desirable to carry out electrochemical experiments where a small amount, or zero, supporting electrolyte is added to solution. For such experimental cases new theoretical models are needed to describe the experiments and allow the experimental electrochemist to interpret results.

### 5.1 The Nernst–Plank–Poisson equations

If migration effects are present in an experiment, Ficks second law alone becomes inadequate to describe mass transport. Instead, the Nernst–Planck equation is used:

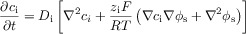
53

where *c*_i_ is the concentration of species i (mol m^−3^), *t* is time (s), *D*_i_ is the diffusion coefficient of species i (m^2^ s^−1^), *z*_i_ is the charge on species i (multiples of the electronic charge), *F* is the Farday constant, *R* is the gas constant, *T* is temperature (K) and 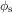
is solution potential (V). This equation describes the mass transport of a solution-phase species in the presence of an electric field.

The solution potential, *ϕ*_s_, is described using the Poisson equation:

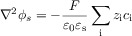
54

where 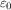
is the vacuum permittivity (F m^−1^) and 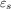
is the relative permittivity of the solvent. These two equations together constitute the Nernst–Planck–Poisson system of equations, and subject to appropriate boundary conditions may be used to model electrochemical experiments in the absence of excess supporting electrolyte.

The Poisson equation can be applied to the simulation of weakly supported electrochemical experiments in different ways. The simplest models assume that the electrical double layer is negligible in extent beyond the electrode, and thus completely exclude it. There are then two main approaches: the electroneutrality approximation and the zero field approximation.

#### 5.1.1 Theoretical treatments neglecting the double layer

The electroneutrality approximation simply assumes the solution to be electroneutral at all points:^130^

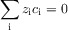
55

A potential profile which satisfies this condition is the mass transport equation and the appropriate electron transfer boundary condition (for cyclic voltammetry this will be the Nernst equation, the Butler–Volmer equation, or Marcus–Hush kinetics, as discussed above). This approximation greatly facilitates analytical solution of the Nernst–Planck equation.^130^ Where no such analytical solutions exist and numerical simulation is required, in the case of transient voltammetry for example, this approximation offers little advantage over other methods which do not make the a priori assumption that the solution is electroneutral at all points.

A more rigorous method is the zero field approximation.^131^ By assuming that the electrical double layer is infinitesimally small, the charge on the electrode surface is completely cancelled in a negligibly small layer of solution immediately adjacent to it. The electrode surface and this infinitesimal layer of solution taken as a whole then has zero charge, and hence zero electric field exists at the electrode surface:

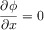
56

This boundary condition may then be used with the Nernst–Planck–Poisson system of equations to numerically generate a potential profile and species concentration profiles across the solution. This method has been shown to be successful in simulating diverse experimental data, at both micro and macro electrodes,^104^,129b–129g,^132^ and is used to obtain theoretical results discussed in sections 5.2 to 5.4.

Neither of these approaches models the electrical double layer, and both assume it is small enough compared to the depletion layer around the electrode to neglect. This approximation is generally valid for electrodes of radius greater than approximately 10 μm. For electrodes smaller than this, the depletion layer around the electrode will approach the size of the electrical double layer, which can then no longer be neglected.

#### 5.1.2 Theoretical treatments including the double layer

Various models exist to simulate voltammetry when a significant double layer is present. The simplest assume that electron transfer takes place at a plane located at a fixed distance from the electrode surface, the plane of electron transfer (PET). This approach leads to the identification of the Levich and Frumkin effects.

Levich predicted^133^ that for a weakly supported system, if 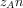
(where 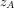
is the charge on the species undergoing electron transfer and 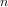
is the number of electrons transferred from the species to the electrode, which is positive for an oxidation and negative for a reduction) is greater than zero, then the reacting species is excluded from the electrode at large overpotentials if a significant double layer is present, lowering the current. This leads to the prediction of peak-shaped steady-state voltammetry. A lowering of the current is also predicted by the Frumkin effect,^134^ which predicts a reduced electrochemical rate constant inside a double layer at large overpotentials, if 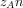
is greater than or equal to zero.

The absence of experimentally observed peak-shaped steady-state voltammetry in many microelectrode systems suggests these pictures are not complete. Rather than assuming electron transfer to occur solely at the plane of electron transfer, Dickinson and Compton^135^ developed a model where electron transfer occurs via a distance-dependent tunneling mechanism across the diffuse double layer. This was shown to dramatically mitigate the Levich and Frumkin effects, and sigmoidal steady-state voltammetry is regained.

### 5.2 How much suppporting electrolyte is needed?

For steady-state voltammetry, it has been proposed that a ratio of supporting electrolyte to electroactive species of 26 is sufficient to ensure full support.^136^ Dickinson et al.^104^ demonstrated that for macroelectrode systems, this is not enough supporting electrolyte and significantly more is needed to avoid the effects of ohmic drop and migration becoming apparent. A ratio in excess of 100 is shown to be required in some cases. As well as large electrodes, it was shown that fast scan rates and slow diffusion of the electroactive species (all of which lead to more transient voltammetry), as well as slow diffusion of the supporting electrolyte all necessitate a higher supporting electrolyte concentration than is sufficient for steady state.

**Figure 17:**
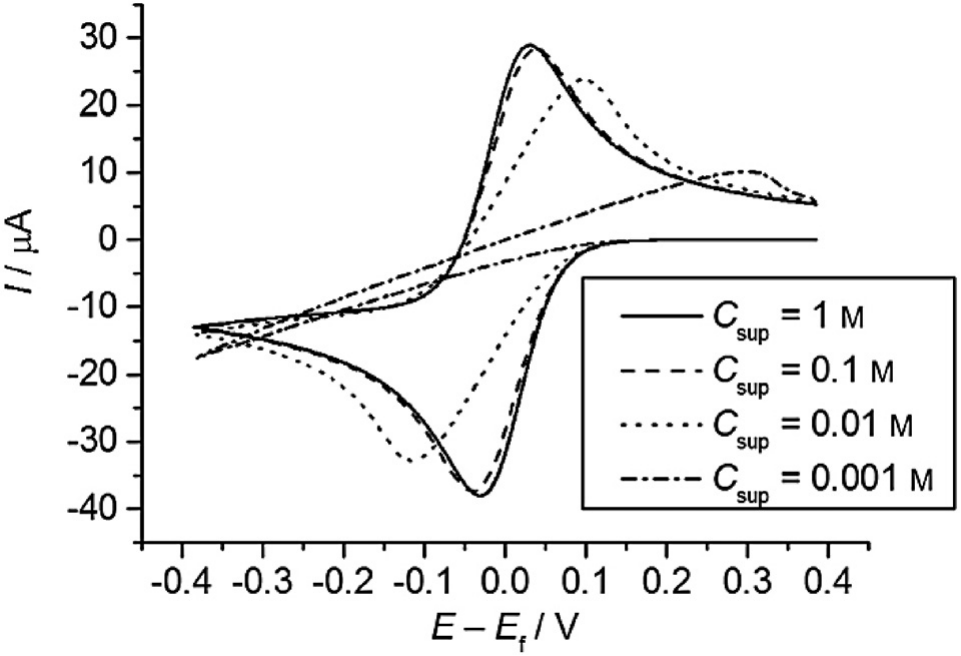
Simulated cyclic voltammograms for a fully reversible one-electron reduction in water at a hemispherical electrode at various support ratios. Parameters: *C*_A_=1 mm, *D*_A_=1×10^−9^ m^2^ s^−1^, *r*_e_=1 mm, *v*=0.5 V s^−1^. All other diffusion coefficients are equal to *D*_A_.

Dickinsons^104^ results are summarised in Figure 17, which shows simulated cyclic voltammograms for the reduction of some neutral species A at a 1 mm radius hemispherical electrode and a scan rate of 0.5 V s^−1^ at various levels of support. The zero field approximation described briefly above was used in the simulations it is seen that even 100 times as much supporting electrolyte as electroactive species is not sufficient in this case to exactly reproduce the fully supported result.

### 5.3 The effects of weak support

Figure 7 also usefully demonstrates some key features of weakly supported voltammetry. As the amount of supporting electrolyte is lowered, the ohmic drop results in a larger peak-to-peak separation and a reduced current.

**Figure 18:**
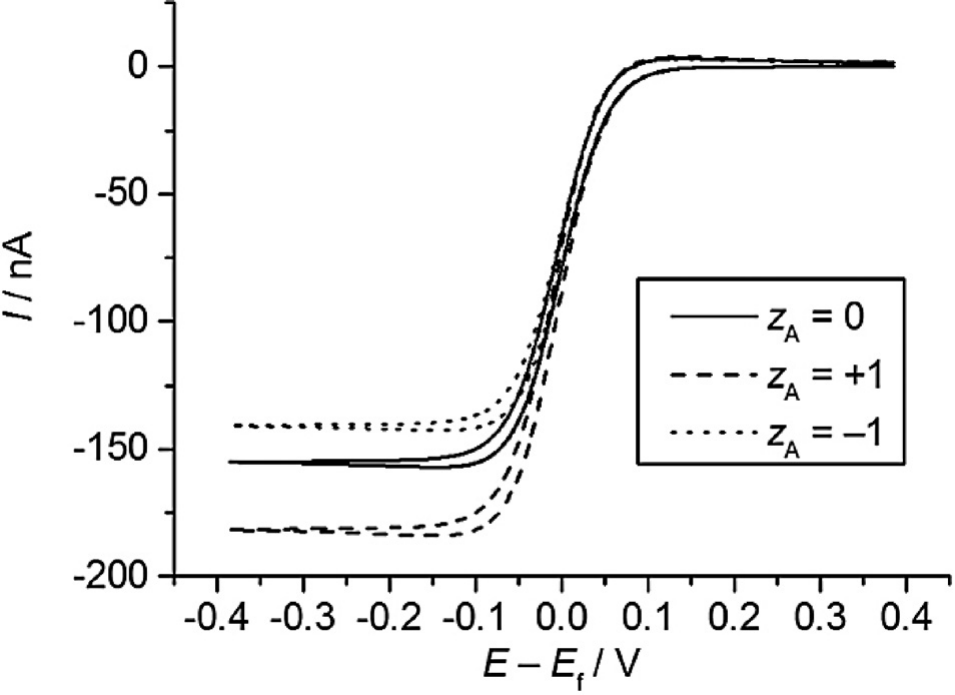
Simulated cyclic voltammograms for a fully reversible one-electron reduction in water at a hemispherical electrode for various charges of electroactive species. Parameters: *c*_A_=1 mm, *D*_A_=1×10^−8^ m^2^ s^−1^, *r*_e_=25 μm, *ν*=1 mV s^−1^, *c*_sup_=1 mm. All other diffusion coefficients are equal to *D*_A_.

The effect of the charge born by the electroactive species was investigated thoroughly by Belding and Compton.^137^ Over the course of a reduction, negative charge is necessarily introduced into the solution, and without a large amount of supporting electrolyte to dissipate this charge it builds up around the electrode. Hence, positively charged species will be attracted towards the electrode, and negative species repelled away from it. This is seen in Figure 18, where the positive species has a higher limiting flux than the neutral species due to its electrical migration towards the electrode. Conversely, the negatively charged species has a smaller limiting flux since it is repelled away from the build-up of negative charge around the electrode. It is worth noting that if the electroactive species is charged, then it and its counter ion can act as supporting electrolyte, resulting in a “self-supported” system. The effect of analyte charge is shown in Figure 18. This figure shows cyclic voltammograms for a fully reversible reduction of some species with a charge of 0, +1, and −1 at a 25 μm radius hemispherical electrode and a scan rate of 1 mV s^−1^, with 1 mm monovalent supporting electrolyte present.

### 5.4 Applications of weakly supported voltammetry

As alluded to above, the effects of migration in weakly supported voltammetry may be used to extract valuable kinetic and mechanistic data unobtainable if experiments are carried out under full support. Three examples of this are discussed below. In general the lack of supporting electrolyte leads to the cyclic voltammetry becoming sensitive to the charge of the reactants, intermediates, and product species.

#### 5.4.1 Comproportionation in the reduction of anthraquinone

If two successive electron transfers occur as in the following mechanism:

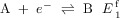
57

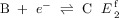
58

then, providing 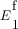
>
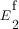

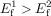
for reductions as shown (or, if the electron transfers were oxidations, 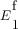
<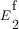) then comproportionation between species A and C becomes thermodynamically (but not necessarily kinetically) favourable:

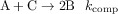
59

Andrieux and Savéant^138^ showed that, under diffusion only conditions, if both electron transfers are fully reversible and all diffusion coefficients are equal, then cyclic voltammetry will be completely insensitive to the presence of comproportionation. Hence diffusion only cyclic voltammetry will be inadequate to establish whether or not comproportionation takes place.

Belding et al.129d investigated the stepwise two-electron reduction of anthraquinone in nonaqueous solvents at both high and low concentrations of supporting electrolyte to determine if the added effects of migration would allow the presense or absence of comproportionation to be determined. Experimental data collected at high support was simulated, and good agreement was seen. Simulations were carried out both in the absence of comproportionation and in the presence of fast comproportionation. The same experiments and simulations were carried out at low concentrations of supporting electrolyte. When comparing simulated data in the presence and absence of comproportionation, differences were seen, with the second reductive peak significantly reduced in size when comproportionation was fast. Comparison of these simulations to experimental data confirmed the presence of fast (*k*_comp_>10^8^ dm^3^ mol^−1^ s^−1^) comproportionation between anthraquinone and its dianion, which is impossible to detect using conventional diffusion-only voltammetry.

#### 5.4.2 Ion pairing in electrochemical mechanisms

It is known^22^,^139^ that the reduction of the diphenylpyrylium (DPP) cation follows an EC_2_ type electrochemical mechanism, with ion pairing of the DPP radical occurring after reduction:

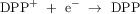
60

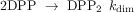
61

The literature reports a large range of values for *k*_dim_ from 2.5×10^7^ dm^3^ mol^−1^ s^−1^ to 2.5×10^9^ dm^3^ mol^−1^ s^−1^.^139^ Barnes et al.129b investigated this reduction at a range of concentrations of tetrabutylammonium tetrafluoroborate supporting electrolyte, and using simulation, were unable to reproduce experimental data across the whole range of supporting electrolyte concentration used. It was found that the experimental peak heights, instead of leveling off at high support (as in Figure 7), continued to decrease (the peak height increased with reducing concentration of supporting electrolyte due to migration of the positive DPP^+^ ion; see above).

To account for this, a further mechanistic step was recognised: a fast ion-pairing equilibrium of the DPP^+^ ion and the tetrafluoroborate anion, BF_4_^−^:

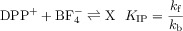
62

where *K*_IP_ (dm^3^ mol^−1^) is the equilibrium constant, defined as the ratio of forward and reverse rate constants, *k*_f_ (dm^3^ mol^−1^ s^−1^) and *k*_b_ (s^−1^) respectively. With this step included in the mechanism, experimental voltammetry was successfully simulated across the whole range of supporting electrolyte concentrations and scan rates used. The concentration of free DPP^+^ was decreased when a large amount of supporting electrolyte was present, and the peak currents therefore decreased. This resolved the literature controversy over the rate of dimerisation, and a value of *k*_dim_=5×10^5^ dm^3^ mol^−1^ s^−1^ was established, with an equibibrium constant for ion pairing of *K*_IP_=35 dm^3^ mol^−1^. These observations were only made possible by the ability to simulate cyclic votlammetry at varying concentrations of supporting electrolyte.

#### 5.4.3 The ECE/DISP1 mechanism

A mechanism often encountered in electrochemistry, especially in the reduction of aromatic halides, is the ECE/DISP1 mechanism:^140^

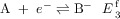
63

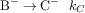
64

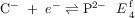
65

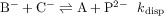
66

If 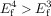
for the reductions as written, then the disproportination step 64 is thermodynamically viable. The source of the second electron transfer may then be either step 63 (the ECE mechanism) or step 64 (the DISP1 mechanism if step 62 is rate limiting, or the DISP2 mechanism if step 64 is rate limiting). While DISP2 is able to be distinguished from the other mechanisms with relative ease,^141^ the discrimination between ECE and DISP1 is difficult.^142^ At high concentrations of supporting electrolyte, careful analysis of voltammetric wave shape over a range of scan rates can in some circumstances be used.129c At steady state, however, it is impossible to distinguish the two mechanisms.

If, however, migration is included as a mode of mass transport through addition of only a small amount of supporting electrolyte, then discrimination may become much easier through analysis of peak heights as a function of supporting electrolyte concentration, as shown by Barnes et al.129a

If the chemical step 62 is such that species B and C have the same charge, as written above (for example, an isomerisation), then species A and C, the two possible sources of the second electron in the mechanism, will undergo migration to different extents (as written in the mechanism above, species A will not migrate at all, and species C will migrate away from the electrode as negative charge is introduced *via* reduction). This means that if the mechanism is ECE, and species C is the source of the second electron, the peak current will be reduced, since species C is repelled from the electrode. If the mechanism is DISP1, and species A is the source of the second electron, migration will not reduce the current since species A is electrically neutral. It is therefore possible, in theory, to assign the mechanism as ECE or DISP1 by carrying out experiments in the presence of both high and low concentrations of supporting electrolyte, and simulating both cases. Only one mechanism should give a consistent value of *k*_c_ over the whole range of supporting electrolyte concentrations used.

Understanding of weakly supported voltammetry has been greatly enhanced in recent years through the use of numerical simulation. Such simulations have been used not only to gain a more fundamental insight into electron transfer processes occurring inside a double layer, but have also been applied to model experimental data. The latter in particular has allowed for greater insight into chemical processes and the discovery of new, sometimes unexpected, information. This demonstrates the power of this technique.

## Section 6: Voltammetry at Rough and Porous Surfaces

Much current experimental electrochemistry is a materials-based activity devoted to the search for electrocatalysts that might assist various technologically important electrode processes including, for example, the reduction of oxygen, the oxidation of methanol, the oxidation of formic acid, and the hydrogen evolution reaction. Voltammetric methods are widely employed to test the success of the electrocatalysts, generally deployed so as to modify the electrode surface, even though the nature of the cyclic voltammetric experiment is rather different from the conditions in which the catalyst is likely to be employed in, say, a fuel cell or battery.

The term ‘catalyst’ implies a change in the rate constant for the process of interest either via a change of mechanism or via the lowering of the activation energy within the same mechanism. To explore the role of an electrochemical rate constant, we consider a simple one-electrode process

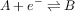
67

where the net rate of the electrochemical process is given by

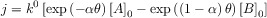
68

and

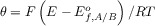
69

**Figure 19:**
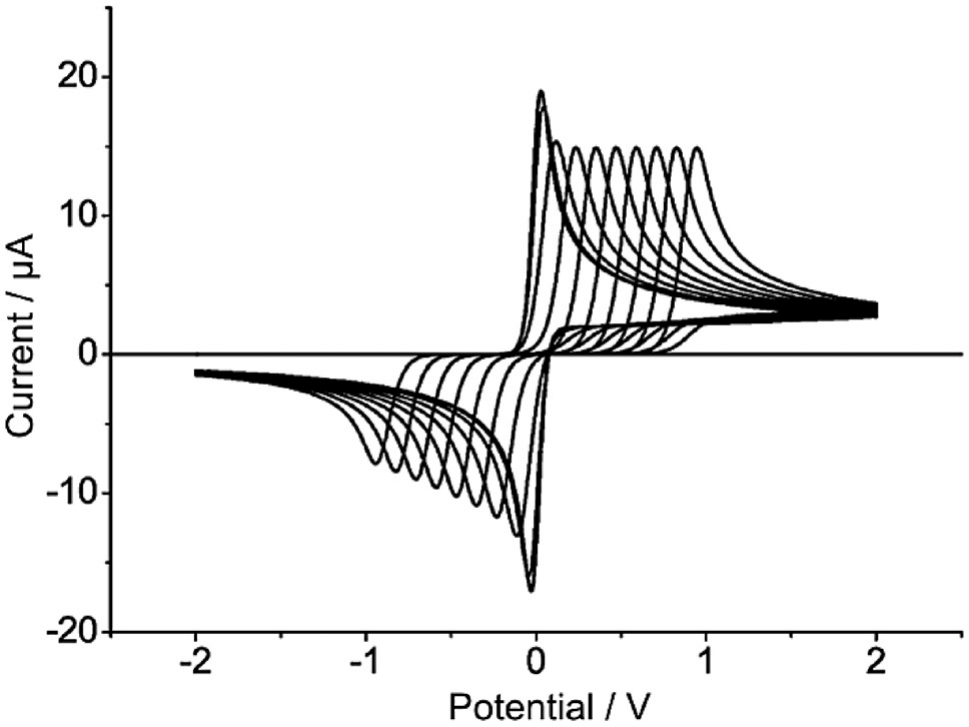
Simulation of one-electron oxidation process. *α*=*β*=0.5, *D*_A_=*D*_B_=1×10^−5^ cm^2^s^−1^, *E*^o^=0, *ν*=0.1 V s^−1^, electrode area=0.0707 cm^2^, *C*^✶^_A_=0.001 m, k^0^ ranges from 1 cm s^−1^ to 1×10^−10^ cm s^−1^. Reproduced with permission from Ref. 143. Copyright 2010, Elsevier.

*k*^0^ is the standard electrochemical rate constant, *α* is the Butler–Volmer transfer coefficient^9^,^143^ and [X]_0_ is the surface concentration of species X. It is well known that as *k*^0^ decreases in size, an overpotential is required to ‘drive’ the electrode process. In terms of cyclic voltammetry this is revealed by an increase in the peak-to-peak separation in terms of the potential as shown in Figure 19 which has been calculated for a typical macroelectrode, radius 0.15 cm, and a voltage scan rate of 0.1 V s^−1^. The value of *k*^0^ ranges from 10^−12^ to 10^−2^ m s^−1^, which spans the range from electrochemically irreversible, through quasi-reversible to fully electrochemically reversible, where the term electrochemical reversibility indicates the speed of the electron transfer (*k*^0^) relative to the prevailing rate of mass transport (*k*_MT_∼*D*/*r* where D is the analyte diffusion coefficient). The Randles–Ševčík equation for the voltammetric peak current of a reversible overall n-electron reduction process is:

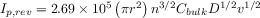
70

whilst that of an irreversible process is:

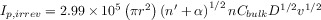
71

where *n*’ is the number of electrons transferred before the rate-determining step (for which the electron transfer is characterised by *α*).

It is interesting to calculate the ratio of the peak currents for reversible and irreversible processes. For the case of *n*=1, *α*=*β*=0.5 and *n*′=0,

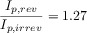
72

as can be seen from Figure 9,^143^ described above. It follows that changing the electrochemical rate constant in this case has a rather tiny effect on the peak current; the effects of changed *k*^0^ (and hence electrocatalysis) are best judged by the peak-to-peak separation, although if the reaction is chemically irreversible, and the processes for which electrocatalyts are most sought are typically of this type, then the absolute potential of a single peak potential might be used but subject to the caveats raised below.

The rather low value of a 27 % decrease in current between the electrochemically reversible and irreversible limits relates to the case of *n*=1. For the case of an *n*>1 process, and assuming that the rate-determining step under irreversible condition is the first electron transfer,

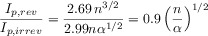
73

This is larger (than 27 %) but still relatively modest.

The insensitivity of the peak current to the rate of electron transfer reflects the fact that the voltammetric peak arises as a competition between the two processes of mass transport (diffusion) and electron transfer. The calculation above assumed semi-infinite diffusion-only conditions, linear diffusion, and a flat and planar macroelectrode. In the case of convergent diffusion to a microelectrode, the enhancement of the current, of course, will be less since under true steady-state conditions, a limiting current will flow, reflecting simply the total number of electrons transferred:

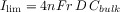
74

It is important to next explore what happens if the electrodes surface remains that of a macroelectrode but is ‘modified’ with a layer of catalyst so as to create a porous structure on the electrode surface, reflecting many of the electrode modification strategies used in energy research.

### 6.1 Semi-infinite diffusion versus thin layer: qualitative insights

**Figure 20:**
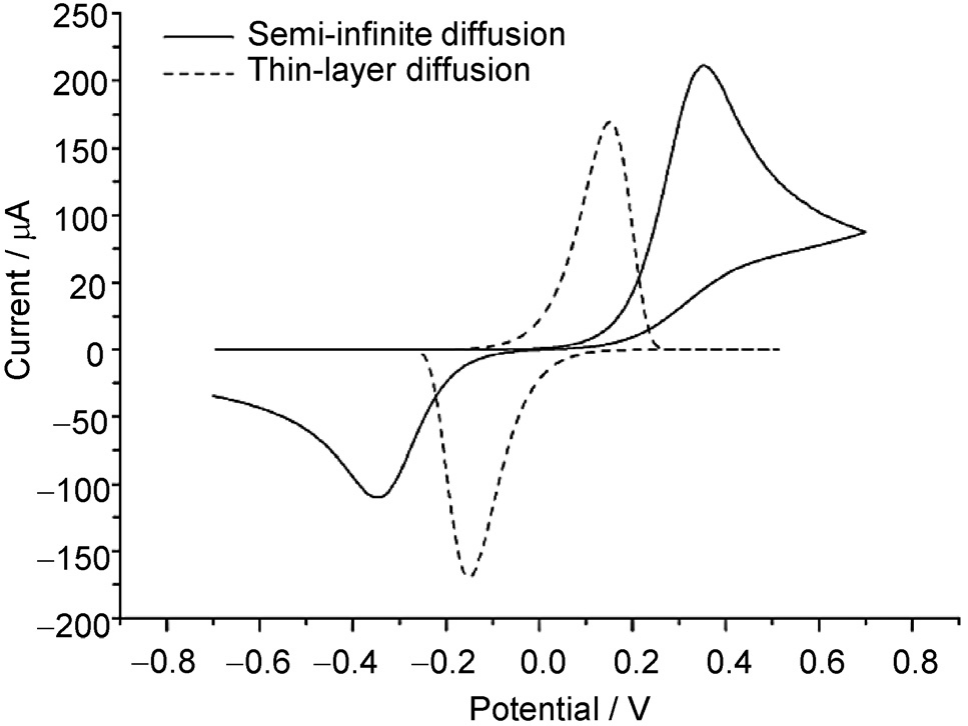
Comparison of linear sweep voltammetry using a semi-infinite and thin layer planar diffusion models. For both models, *k*^0^=10^−4^ cm s^−1^; *D*=10^−5^ cm^2^ s^−1^; *ν*=0.1 V s^−1^; *c*=10^−6^ mol cm^−3^. Semi-infinite diffusion electrode area, *A*=1 cm^2^; thin-layer area, *A*=30 cm^2^; thickness, *l*=1 μm. Reproduced with permission from Ref144a. Copyright 2008, Elsevier.

A particular important case is when a macroelectrode is modified with a conductive porous layer made, for example, of carbon nanotubes, graphene, or nanoplatelets. In this situation, the mass transport of solute to the conductive surface (assumed to be both the substrate electrode and the conductive, modifying layer) arises from two components: First, semi-infinite diffusion from the solution bulk to the surface of the porous layer and, second, a component due to diffusion transport within the porous layer.^144^ If the packing density and thickness of the porous layer is suitably large (but typical) then the transport within the porous layer can be approximated as a ‘thin layer’ in which the distance diffused by the solute in order to reach a location where it can be electrolyzed is short as compared to that which occurs under semi-infinite diffusion conditions, and hence, the (diffusional) overpotential is markedly reduced reaching, in the limit, apparent reversible ‘thin-layer’ behavior. Figure 20 shows the effect: it compares cyclic voltammetry for a thin-layer system (area 30 cm^2^, thickness 1 μm) with that seen for semi-infinite diffusion from a system with *D*=10^−5^ cm^2^ s^−1^ and *C*_bulk_=1 mm for a standard electrochemical rate constant of 10^−4^ cm s^−1^ (and *α*=0.5). Note the much reduced peak-to-peak separation for the thin-layer case with the contrast from semi-infinite diffusion arising solely from the altered mass transport.

The shift in the peak potential under increasingly thin-layer conditions will ultimately (as the distance required for diffusion is steadily reduced) tend to a limiting value where the potential corresponds to that of the formal potential of the couple of interest. This observation explains the fact that the modification of an electrode with an electrochemically conductive porous layer can resolve otherwise overlapping voltammetric peaks. This has potential analytical value144b and can simply arise as a consequence of the altered mass transport; it is not necessary to invoke changed electron transfer kinetics.

Finally we point out that under extreme conditions it should be noted that it is possible to see two peaks resulting from a single A/B redox couple: one is a thin-layer signal and the other that arising from semi-infinite diffusion to the surface of the porous layer.144d

### 6.2 Carbon nanotube and other modified electrodes

**Figure 21:**
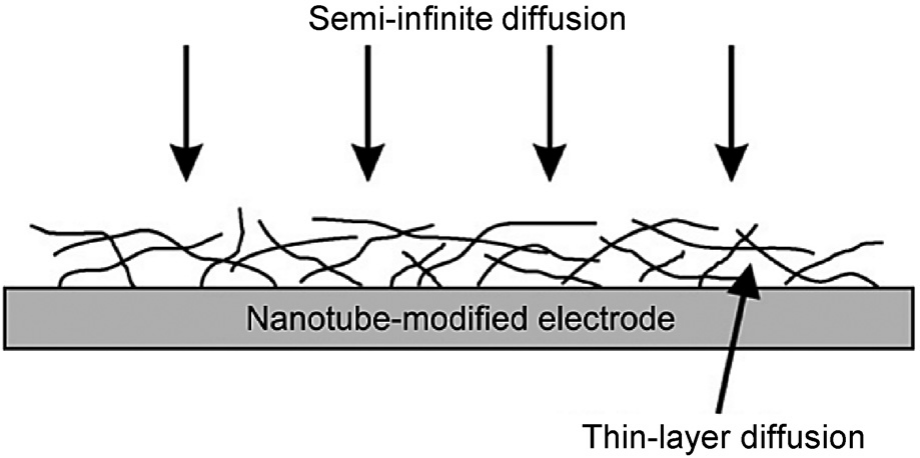
Schematic of the two types of diffusion that contribute to current at a carbon-nanotube-modified electrode. Reproduced with permission from Ref. 144a. Copyright 2008, Elsevier.

The effects predicted for thin-layer versus semi-infinite diffusion voltammetry are consistent with observations made using a range of systems.144a–144c In particular, there has been very considerable work in using carbon nanotubes (or chemically modified nanotubes) to create porous layers on the surface of electrodes. The observed voltammetry is consistent with the ideas outlined in the previous section (refs. 144a–144c and refs. therein). Figure 21 shows the basic model in which the trapped products of analyte-containing solution act as small thin-layer cells. A key indicator of this behavior is the observation that the peak currents flowing associated with the thin-layer behavior can be significantly larger than those seen for semi-infinite diffusion at an electrode of the same geometric area. Note, as discussed above, such large enhancements are not understandable in terms of altered electrode kinetics per se. The work of Henstridge et al.144b contains tables of examples of CNT and other modified electrodes in which the thin-layer behavior may operate. Similarly Kozub et al.^143^ report ‘electrocatalytic’ systems developed allegedly for the detection of nitrite.

### 6.3 Effective heterogeneous rate constants for rough and porous surfaces

**Figure 22:**
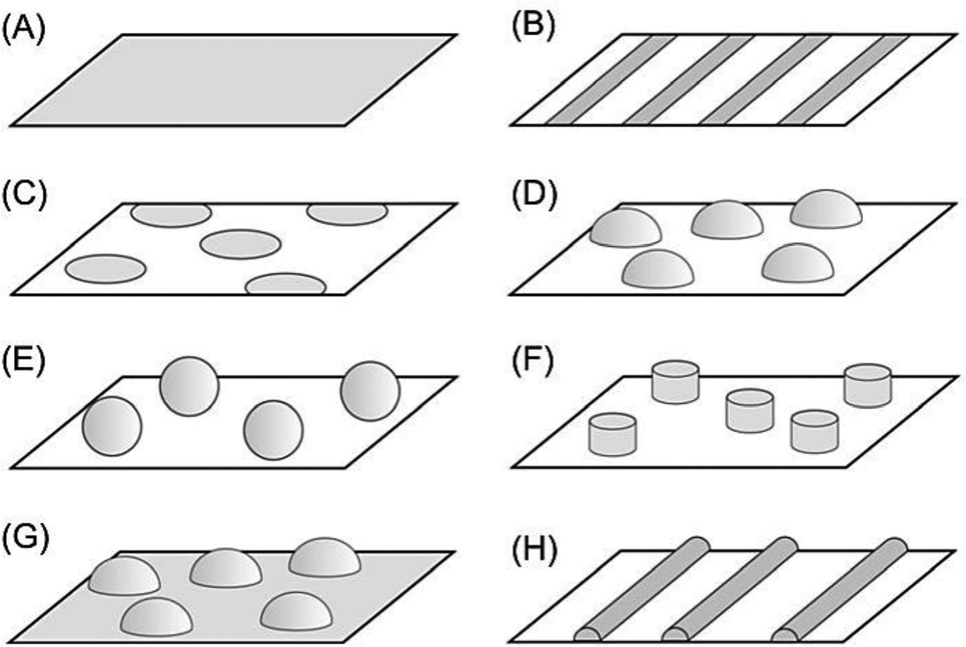
Electrode geometry types discussed in Section 6.3. Shaded regions are electroactive, white areas are inactive. Reproduced with permission from Ref. 145b Copyright 2008, Elsevier.

**Figure 23:**
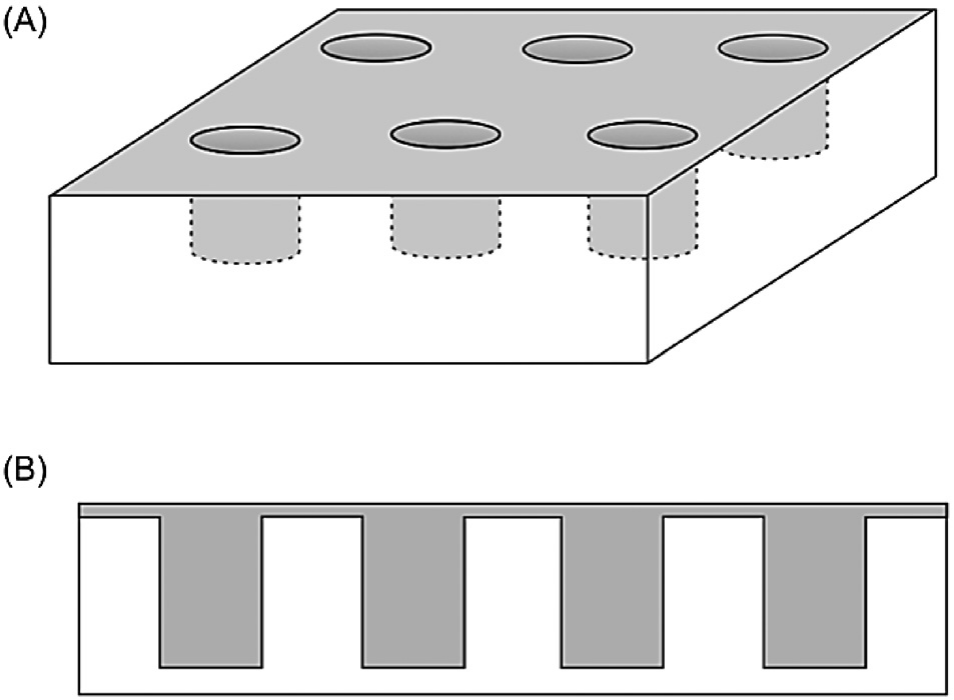
A) Schematic of a porous surface; B) side on view. Reproduced with permission from Ref. 145a. Copyright 2014, Elsevier.

Numerical simulation^145^ has been explored to identify the effective standard electrochemical rate constant for both rough and porous surfaces using surface morphologies such as those shown in Figure 22 and 23. The former models a dense, but less than monolayer array of nanoparticles whilst the latter approximates a porous surface. In the former case, the apparent electrochemical rate constant (inferred from the peak potential of the voltammetry) was seen to vary according to

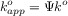
75

where *k*^o^ is the true electrochemical rate constant, and 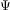
is the ratio of the electroactive surface area to the geometric surface area.145b In this case the peak potential is given by

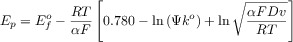
76

for a one-electron process. Note that this relationship assumes that the adjacent particles are sufficiently close together, that on the timescale of the experiment the diffusion field is normal to the place of the bulk electrode surface; that is to say, the diffusion fields of the particles are heavily overlapped, which is usually the case except for very dilute coverages.

**Figure 24:**
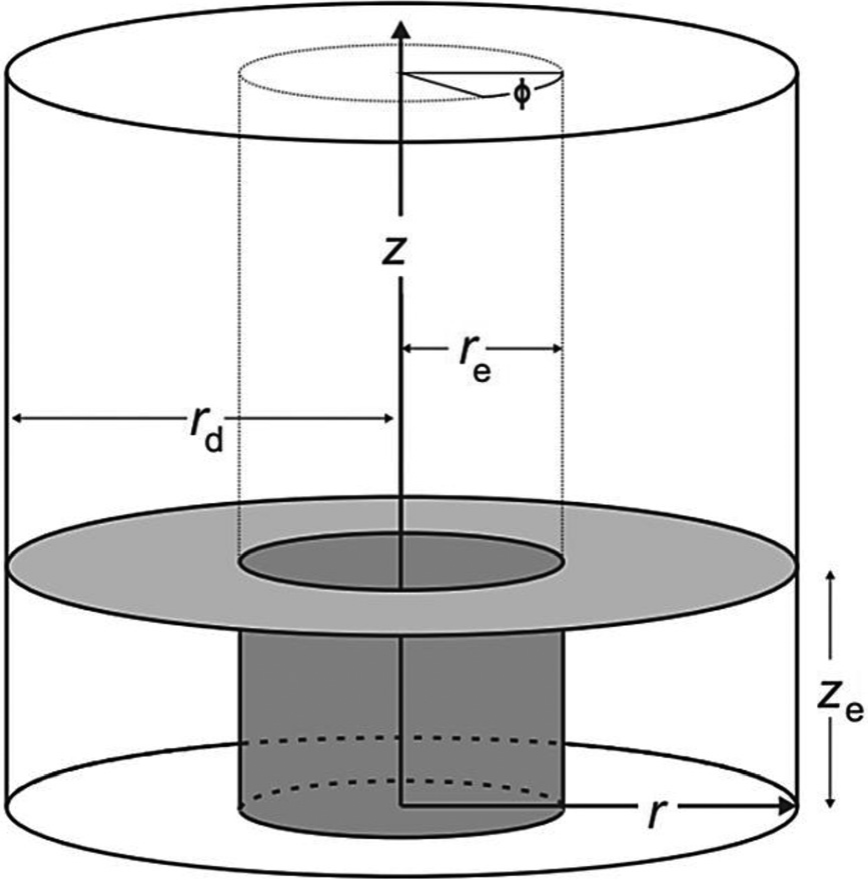
Unit cell and coordinate system for a porous surface with coordinates *r*, *z*, and *ϕ*. Reproduced with permission from Ref. 145a. Copyright 2014, Elsevier.

In the case of a porous surface,145a

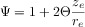
77

where *z*_e_ is the depth of the pores, *r*_e_ is their radius and 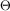
is the porosity defined by,

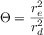
78

where *r*_e_ and *r*_d_ are shown in Figure 24 as defined by the diffusion domain approximation to the electrode surface.^145^ The effects of the roughness and porosity are illustrated in the following subsection but an important general extension relates to the use of rotating disc electrodes to extract the number of electrons (*n*) transferred and the electrochemical rate constant by measuring the transport limited current as a function of disc rotation speed and applying the Koutecky–Levich equation.^146^ It was found that this analysis gives correct values of *n* but the apparent, rather than true, rate constant as defined above.^146^

### 6.4 Evaluating oxygen reduction catalysts

The results of the preceding section have been applied in particular to a consideration of nanoparticle-modified electrodes for oxygen reduction catalysis.^147^ Such evaluations often involve simply a measurement of a current at a fixed potential. Simulations of the type reported in the previous section clarified that such currents are sensitive to the surface coverage of nanoparticles without any change in the fundamental kinetics or thermodynamic parameters, even if the voltammetry shows that the reduction operates under full diffusional transport control. The need for caution in the evaluation of catalysts in the manner discussed was evident and an essential need for characterising coverage, porosity, and particle size demonstrated for establishing authentic electrocatalytic character.

### 6.5 Voltammetry at thin-layer, nanoparticle-modified electrodes

**Figure 25:**
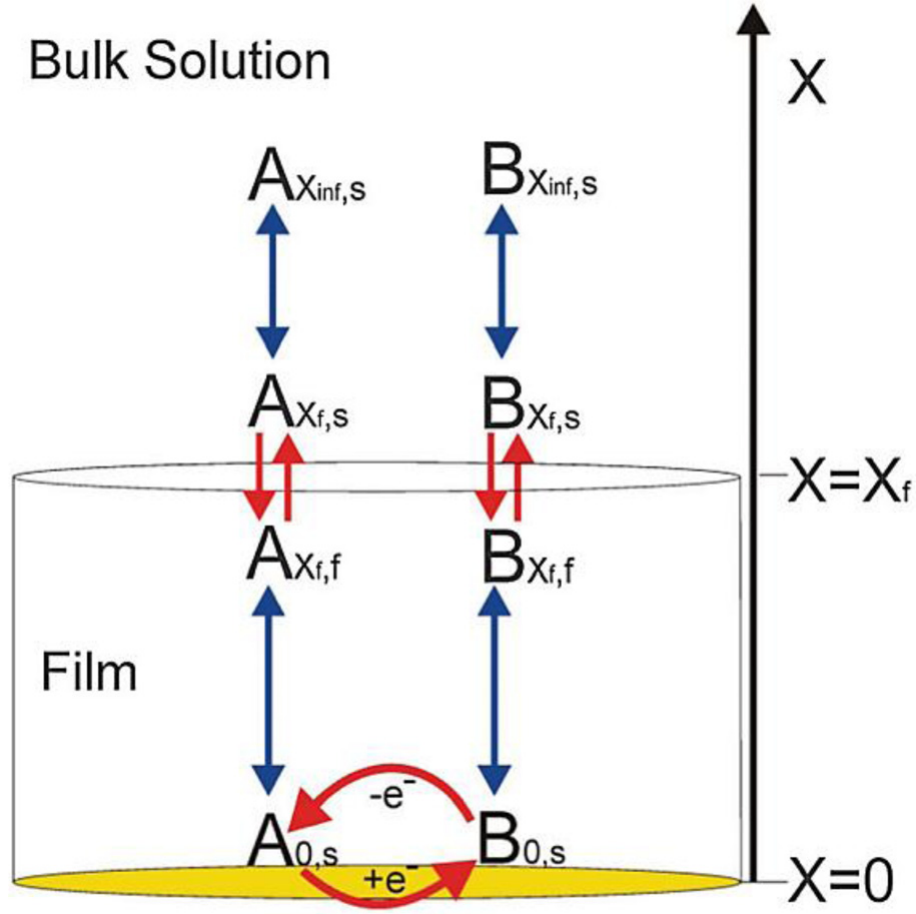
Schematic design of the thin-layer model used in this article, where *x* is the distance perpendicular to the electrode surface (*x*=0). The film thickness is *x*_f_. Reproduced with permission from Ref. 148. Copyright 2014, PCCP Owner Societies.

The previous section concerned electrodes modified with electrochemically conductive layers or films. A more general situation has been modelled using the scheme summarised in Figure 25, in which the modifying layer itself is nonelectroactive but changes the voltammetric response by virtue of altering the solubilites and diffusion coefficients of the electroactive species within the layer as compared to the bulk solution. The electron transfer in this scheme is limited to the surface of the substrate electrode. Both cyclic voltammetry^148^ and electrochemical impedance spectroscopy (EIS)^149^ were modelled; it was established that the illusion of altered electron-transfer characteristics could be generated in each case merely by altered diffusion or solubility. The difficulty of unambiguously modelling the response was noted.

## Section 7: Nanoparticle Voltammetry

As discussed in the previous section, alteration of the electrode surface structure can lead to *apparent* changes in the electron transfer kinetics of an electrochemical reaction; this arises due to changes in the mass-transport regime local to the interface, and alters what would classically be referred to as the ‘*diffusional overpotential*’.^150^ In light of this insight, this section focuses on understanding the voltammetry of electrodes modified with submonolayer coverages of nanoparticles, how *true* electron transfer rates may be extracted via simulation from the experimental data, and how the diffusion field influences the stripping voltammetry of nanoparticles. Using the strategy outlined within this section, it is now possible to rigorously investigate the presence or absence of ‘nano-effects’ arising from the use of novel nanomaterials in voltammetric experiments. In the final part of this section, an alternative technique for studying nanoparticle electrochemistry is highlighted. This new technique referred to as ‘nano-impacts’ exhibits a number of advantages over more conventional electrochemical investigative techniques.

### 7.1 Reactions on nanoparticle-modified surfaces

**Figure 26:**
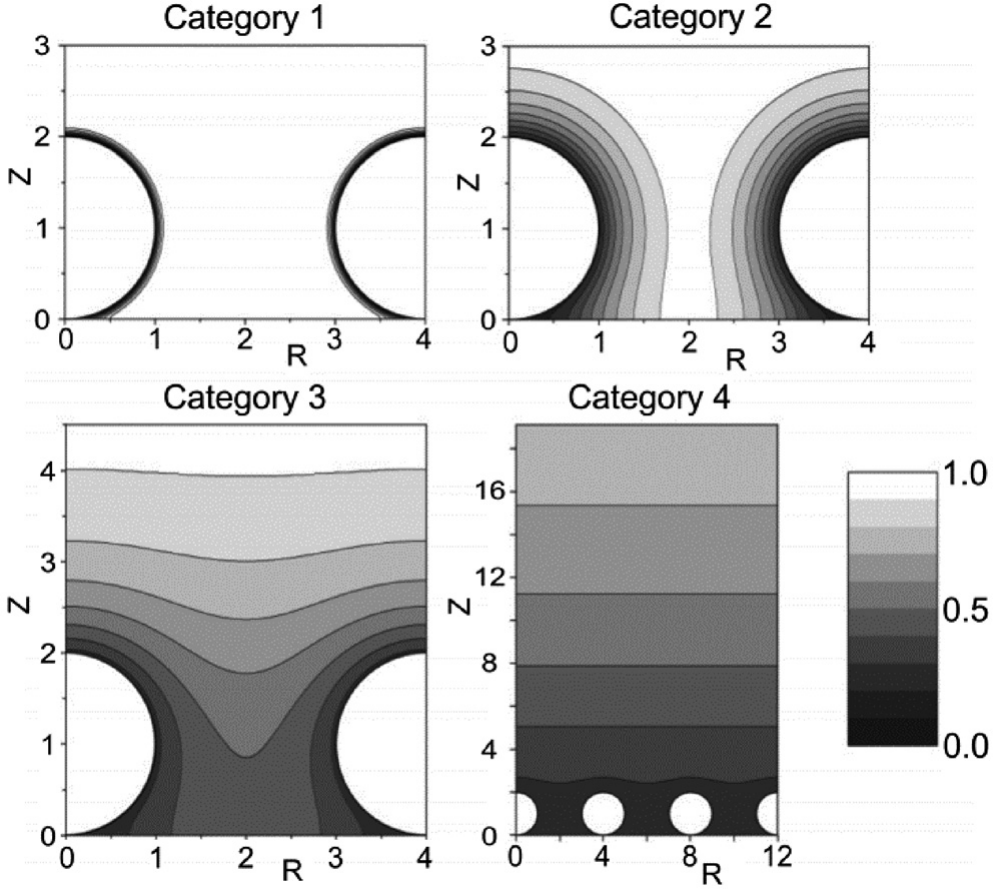
Simulated concentration profiles at a diffusion domain containing a spherical particle. Category 1: *σ*=1000. Category 2: *σ*=10. Category 3: *σ*=1. Category 4: *σ*=0.01, where *σ* is the dimensionless scan rate. Concentration profiles were taken at the linear sweeps peak potential. Reproduced with permission from Ref. 151b. Copyright 2007, American Chemical Society.

For an array of nanoparticles supported upon an electrochemically inert electrode substrate, the mass transport to and from the nanoparticulate surface, and hence the voltammetric behaviour of the electrode, depends upon: the nanoparticles size and morphology, the diffusion coefficient of the analyte and product, the experimental (voltammetric) time scale, and the interparticle separation (nanoparticle surface coverage).^151^ Assuming the substrate electrode is macroscopic in dimensions, then the diffusion regime may be categorised into four cases. Importantly, during the course of a voltammetric scan, the prevailing diffusion regime will likely transit from one case to another; consequently, insight into the voltammetry of such systems is best achieved through simulation. Figure 26 schematically outlines the four diffusional cases or categories.

First, at very short times, the diffusion profile at each individual nanoparticle is linear; this situation occurs for times of the order of *r*_NP_^2^/*D* (commonly ∼1 μs, where *D*∼10^−10^ m^2^ s^−1^ and r_NP_∼10 nm). As the experimental time increases, the diffusion layer grows in accordance with the Einstein equation (*δ*∼(2 *Dt*)^0.5^); overlap between the diffusion layers of adjacent nanoparticles occurs when the diffusion layer thickness *δ* is comparable to the interparticle separation. Case 2 arises for situations where the nanoparticle diffusion layers do not significantly overlap, but the experimental time is greater than *r*_NP_^2^/*D*. Here, the mass transport to each nanoparticle is convergent and can be considered independently of adjacent particles. For an isolated sphere on a surface, the steady-state diffusion-limited current is given by;^152^

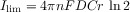
79

At longer experimental times, the diffusion layers of adjacent particles will overlap strongly. Under such situations, classified as case 4, the mass transport to the whole electrode surface is linear, resulting in a macroelectrode response with an associated *apparent* electrochemical rate constant. The magnitude of this rate constant is a function of the nanoparticle surface coverage.145b Transition between isolated nanoparticle diffusion layers (case 2) and strongly-overlapping layers (case 4) leads to case 3. Case 3 is a situation regularly encountered with modified nanoparticle surfaces where the diffusional layers are partially overlapping and can only be approached through simulation. A fifth diffusional case may also be considered as an extension of the above model; this case arises for the situation in which the dimensions of the supporting electrode is only of the order of microns.^153^ Under this case, the mass transport to the whole array is convergent. This category may be viewed partially as a breakdown of the diffusion domain approximation used in macroelectrode simulation models and is notably of significance in application to some SECM experiments.^154^

Due to the complex interplay between the electrode surface geometry and the diffusion profile as outlined above, extraction of physically significant kinetic data from modified electrodes is a nonfacile problem. To experimentally evidence this point, the one electron reduction of chromium(III)^155^ has been studied at a silver-nanoparticle-modified electrode and a silver macroelectrode, demonstrating clearly how the voltammetric response varies as a function of nanoparticle surface coverage. It should also be commented that normalisation of voltammetric results relative to the total electroactive area is also insufficient to, in many cases, allow even a *qualitative* comparison of data.145b Theoretically this conclusion has been specifically validated in relation to the oxygen reduction reaction at nanoparticle-modified surfaces.^147^ Moreover, experimentally, it has been demonstrated how for sparse coverages of platinum nanoparticles on an electrochemical interface, the situation is further complicated by the release of the hydrogen peroxide as an intermediate in the reduction process.^156^ As discussed within Section 1, this alteration of the electrochemical mechanism, arising from the enhancement of the mass transport to and from diffusionally isolated nanoparticles has large implications not just for the industrial use of such nanomaterials as catalysts, but may also be of importance in the context of the toxicity of these substances within biological systems.

**Figure 27:**
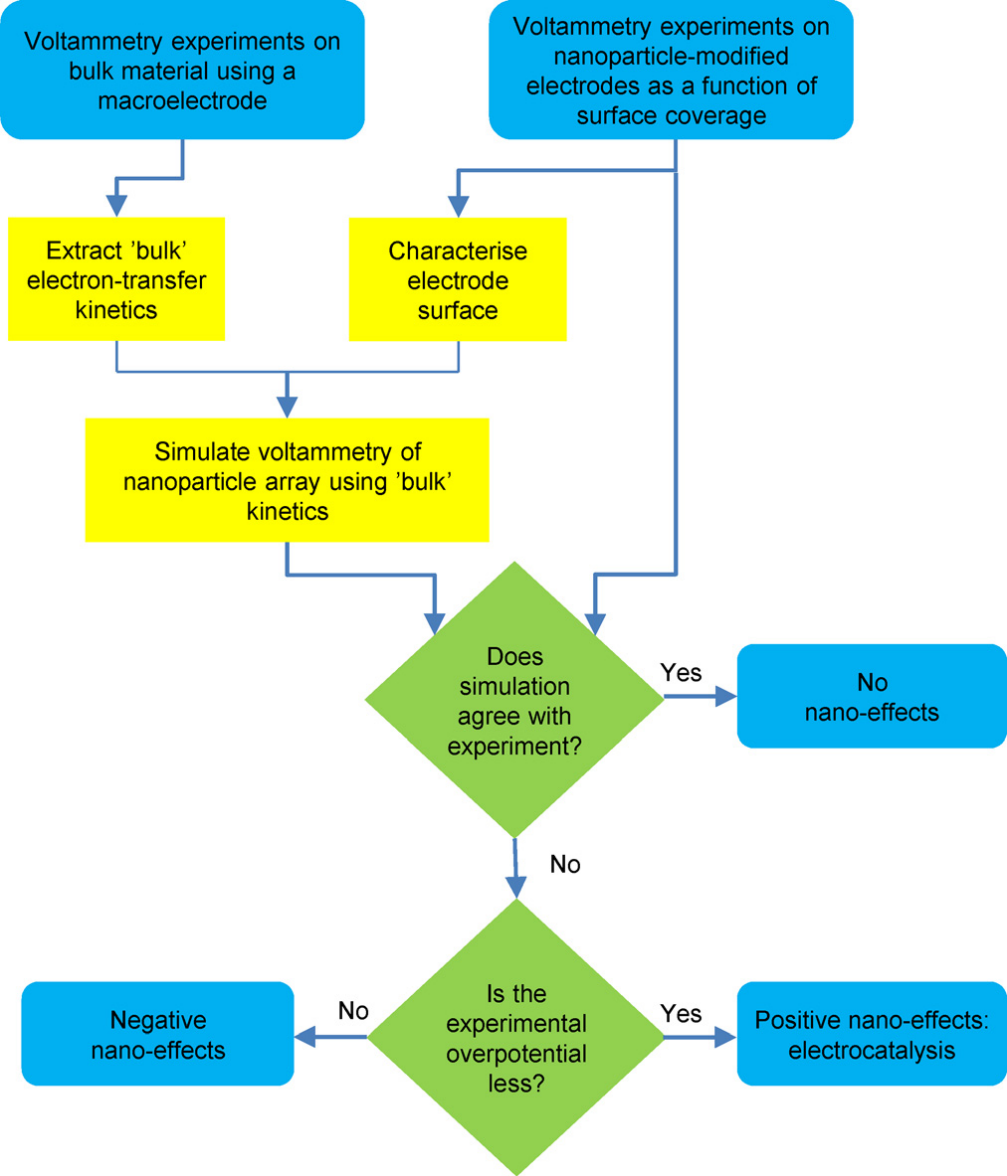
Flow diagram showing the proposed strategy for delineating kinetic and mass-transport effects allowing the detection of authentic nanoelectrocatalysis.

Figure 27 outlines a general strategy for the combined experimental and computation study of electrocatalytic processes at electrodes modified with ensembles of nanoparticles. This methodology allows the influence of the mass transport and the interfacial electron-transfer kinetics upon the voltammetry to be clearly delineated.^157^ Briefly, the approach requires characterisation of the electrode in terms of particle size, aggregation, and separation, allowing the voltammetric response to be simulated using the kinetic parameters obtained using a macroelectrode. From comparison of the simulated ‘bulk’ response and the experimentally recorded data, it is possible to determine if the kinetics have been altered while fully accounting for the diffusional mass transport of the material. From this analysis three outcomes are possible. If the nanoparticle array simulation using the ‘bulk’ kinetics is in good agreement with the experimentally recorded data for the nanoparticle array, then the conclusion must be that there is no evidence of a nano-effect associated with using the nanomaterial. Conversely, if the simulated voltammetric response differs from that found experimentally, then the kinetics of the electrochemical reaction must have been altered through the use of the nanomaterial. This alteration in the kinetics may lead to an *increase* in the overpotential required to drive the reaction; hence, for such situations, the nanomaterial is *less* catalytic than the bulk material, and one has evidenced a ‘negative’ nano-effect. Alternatively, if the overpotential required for the electrochemical reaction is *decreased* as compared to the simulated result then it can be confirmed that the use of the nanomaterial has led to an *authentic* nano-effect. This procedure was applied to the experimental study of arrays of gold nanoparticles ranging in size from 20 to 90 nm in diameter. From these experiments, it was confirmed that for nitrite electro-oxidation, no alteration in the kinetics is observed between the use of gold nanoparticles and a gold macroelectrode.^157^ Conversely, the electro-oxidation of l-ascorbate was shown to exhibit true nanocatalytic effects. The origins of these differences were ascribed as likely being due to the l-ascorbate oxidation involving adsorbed intermediates.^157^ Finally, this same methodology has been applied to the oxygen reduction reaction and the hydrogen evolution reaction, where for small gold nanoparticles (1.9 nm diameter), the electrochemical processes were found to be significantly hindered as compared to the kinetics recorded on the macroelectrode.^158^ This is a prime example of how decreasing the size of the nanoparticles has led to a ‘negative’ electrocatalytic effect, likely resulting from the changed reaction intermediate adsorption on the gold surface.

### 7.2 Stripping voltammetry: the direct oxidation and reduction of nanoparticles

The above examples focus on the situation in which the nanoparticle-modified electrode is utilised to study the electro-catalytic properties of the nanoparticles towards a given redox process. However, the diffusional cases outlined above must also be considered when studying the direct oxidation or reduction of the nanoparticles themselves—as is commonly undertaken during the course of an electrochemical nanoparticle stripping experiment. Such nanoparticle stripping experiments are purportedly a facile route to directly study the possibly altered thermodynamics of the metallic nanoparticles^159^ and their interactions with the electrode substrates.^160^ However, problems arising in such experimental setups relating to partial oxidation or reduction of the nanomaterial has been previously noted—a problem that is circumvented by the use of nano-impacts experiments (see final part of this section for further discussion).^161^

In nanoparticle stripping voltammetry, for the case in which the product of the nanoparticle oxidation or reduction is soluble, the diffusion layer associated with the redox product must be considered. Both simulations^162^ and analytical expressions^163^ have been previously provided demonstrating clearly how, for the stripping of nanoparticles from an electrode surface, the observed peak potential varies as a function of the total amount of electroactive material on the electrode surface, the inter-particle distance and the voltammetric scan rate. Moreover, due to the sensitivity of voltammetry towards the mass-transport regime local to the interface, stripping voltammetry can also be used as a route to indirectly evidence agglomeration of the nanoparticles upon the electrode surface.^163^ In accord with nanoparticle electrocatalysis studies, care must be taken in the analysis of the voltammetric response, accounting for the influence of diffusion prior to ascribing the altered peak positions as relating to changes in the thermodynamics of the nanoparticles. Finally, the use of microelectrodes in stripping voltammetry (an analogous diffusion regime to Case 5 discussed above) may provide one route by which true nanoparticle thermodynamic effects may be more readily investigated, aided by the relatively well-defined steady-state diffusion.^164^

**Figure 28:**
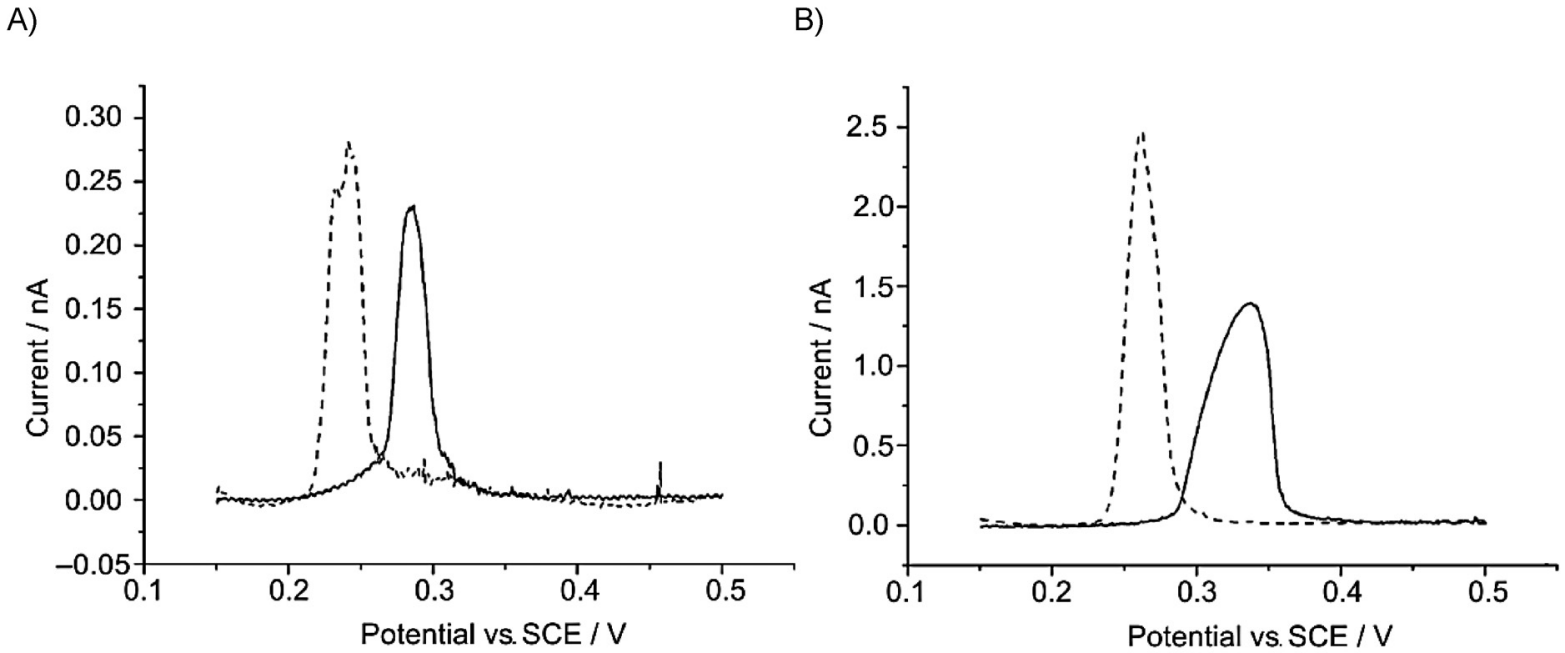
Anodic stripping voltammetry for small (*r*_np_=3.7 nm, dashed line) and large (*r*_np_=13.5 nm, solid line) silver nanoparticles supported on a micro carbon-fibre electrode (*r*_0_=5.5 μm): A) low surface coverage of silver (1.6×10^5^ mol m^−2^) and B) high surface coverage (1.6×10^4^ mol m^−2^). Scan rate: 50 mV s^−1^ and supporting electrolyte of 0.1 m NaClO_4_. Reproduced with permission from Ref. 164. Copyright 2014, Wiley-VCH Verlag GmbH & Co. KGaA, Weinheim.

Figure 28 depicts the stripping voltammetry for two sizes of nanoparticles at two different surface coverages from a carbon-fibre microelectrode (r_0_=5.5 μm), where the observed shift is consistent with a change in the thermodynamics due to the influence of the altered surface energy. Note that the observed peak potential also varies in both cases as a function of the total surface coverage of silver. Not all nanoparticle redox reactions result in the dissolution of the nanoparticle (though there will be a corresponding change in the nanoparticle morphology). One example of such a case is the oxidation of silver in the presence of a halide. Depending on the halide concentration, the electrochemical oxidation will likely lead to the formation of surface bound silver halide.^165^ A secondary example would be the electrochemical formation of metal oxides.^166^ The voltammetric response of such systems is highly complicated; first and foremost, the formation (or solubility) constant between the formed nanoparticle ion and the solution-phase counter-ion serves to alter the thermodynamics of the redox species in accordance with the Nernst equation.^167^ Second, the reduction or oxidation may exhibit complex behaviour such as following a nucleation growth mechanism.^168^ Third, speciation of the products may vary as a function of counter-ion concentration.^169^ Finally, in some cases the reaction may be limited by the mass transport of the counter-ion to the electrochemical interface. Consequently, again when studying these systems, care must be taken when ascribing any alteration in the stripping peak potentials as relating to altered thermodynamics or ‘nano-effects.’ This is especially true due to the fact that the nanoparticle capping agents have been shown to influence the observed stripping voltammetry.^161^ As an interesting aside, the strength of the binding of silver ions to halides and the corresponding Nernstian shift in the stripping peak yield an analytically useful route to their detection via the use of nanoparticle stripping voltammetry.^167^

From the above discussion in the last two sections, it is clear that although not unsurmountable, the interpretation and measurement of physically significant values from the electrochemical response of electrodes modified with ensembles of nanoparticles is inherently challenging. Consequently, there is a desire to find methods by which nanoparticles and their properties can be studied *individually*. One route through which this has been achieved is with the use of so-called ‘nano-impact’ experiments as will be discussed in the next and final section.

### 7.3 ‘Nano-impacts’

In ‘nano-impact’ experiments the nanoparticles are suspended within an electrolyte and randomly, by virtue of Brownian motion, collide with a potentiostated microelectrode.^170^ Upon impact the nanoparticle makes electrical contact, and assuming a suitable potential is held upon the electrode, either the direct electrochemistry of the nanoparticle or a catalytic process of interest may be induced. The resulting current yields direct and significant information regarding the interfacial redox processes occurring at individual particles.

The use of ‘mediated’ nano-impacts has been demonstrated for a variety of systems.^171^ One important factor in such experiments is that the electrode receiving the nanoparticles is inert, such that the reaction of study does not occur on its surface in the absence of the nanoparticle. This requirement has led some researchers to use mercury as the ‘inert’ electrode substrate.^172^ However, problematically in the presence of *trace* chloride, mercury is readily oxidised to form calomel nanoparticles.^173^ Once formed, these calomel nanoparticles are easily reduced at the mercury electrode and can lead to results which may be misinterpreted. Consequently, in many cases, carbon electrodes are found to be more suitable candidates for the supporting electrode material. A notable exception to this is an early example of the use of nano-impacts for study of the oxidation of hydrazine on platinum nanoparticles at a gold electrode.^174^ The difference in the electron-transfer rate on platinum versus gold is sufficient to allow the process to be solely studied upon the impacting platinum nanoparticle. It should, however, be noted that for this system, the presence of hydrazine in solution does cause significant aggregation of the particles.^175^

**Figure 29:**
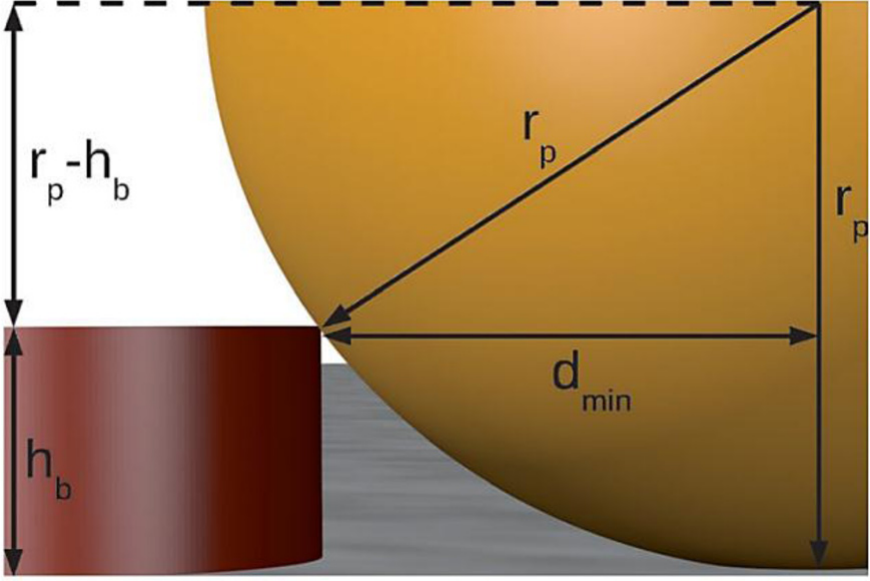
Diagram for the calculation of the minimal distance, *d*_min_, to a blocking molecule, at which a nanoparticle can touch the electrode surface (impact). Reproduced with permission from Ref. 177 Copyright 2014, Wiley-VCH Verlag GmbH & Co. KGaA, Weinheim.

One significant observation for impact experiments is that the recorded collision frequency is not uncommonly below that theoretically predicted.171b This observation has previously been explained solely in terms of solution-phase agglomeration/aggregation.^175^ However, other causes for such effects need to be considered. In many experimental cases the working electrodes used tend to be micron sized wires sealed in glass. Consequently, if the nanoparticles of study also adhere to the inert (glass) substrate surrounding the electrode, then the frequency of observed nanoparticle impacts may be significantly reduced due to diffusional shielding.^176^ A further issue arises upon consideration of the dimensions of nanoparticle as compared to a molecule. As a result of the geometrical constraints of an impacting nanoparticle, the ‘nano-impact’ experiment will be inherently more sensitive to the presence of adsorbing and blocking organic media than conventional molecular redox probes.^177^ Figure 29 depicts a simple geometric model showing how the presence of surface-adsorbed species leads to efficient blocking of the electrode surface towards nanoparticles due to the magnitude of the minimum distance (*d*_min_) of an impacting nanoparticle to the blocking molecule. The magnitude of this minimum distance of approach depends upon both the radius of the nanoparticle (*r*_p_) and the height of the adsorbed species (*h*_b_). Hence, the observed decreased impact frequency may not just be related to agglomeration or aggregation of the nanoparticles in solution, but may also arise due to factors relating to the electrode and the electrode design itself.

Although each individual impact experiment (chronoamperogram) is studied at a fixed potential, performing repeat experiments at differing potentials can yield significant information regarding the kinetics of the catalytic process on the nanoparticle.^178^ Comparison of the kinetics for the reduction of protons at an impacting gold nanoparticle compared to that found from more conventional ensemble measurements led to the conclusion that the process was significantly slower on the individual impacting gold nanoparticle.^179^ This apparent decrease in the kinetics and the notable fluctuations observed in the current during the course of a nanoparticle impact was interpreted as likely indicating a contact resistance between the electrode and the impacting nanoparticle. Consequently, although the presence of the nanoparticles in solution can be evidenced from their catalytic impacts at an electrode, the full potential of this method has yet to be realised.

Apart from the study of the *catalytic* response of impacting nanoparticles, in analogy with nanoparticle stripping voltammetry, the direct redox of the impacting nanoparticle may also be studied. The first example of such an experiment was the in situ detection of silver nanoparticles.^180^ Upon impacting an electrode with a suitably anodic potential, the nanoparticles were oxidized, resulting in small spikes of current. Through Faradays first law, the magnitude of these spikes can be related directly to the total number of atoms contained within the individual impacting nanoparticle, hence providing measurement of its size.^161^,^181^ Furthermore, the frequency of the impacting spikes can yield information regarding the concentration of the nanoparticles in solution.^182^ This new nano-metrology technique has been successfully applied to a host of different nanoparticle materials including other metals,^183^ metal oxides,^184^ carbon fullerenes,^185^ organic nanoparticles,^186^ and liposomes.^187^ Work has also demonstrated that the technique is suitable for use in complex media such as sea water, highlighting its potential use in environmental analysis.^188^ To this end the use of carbon-fibre microcylinder electrodes has been advocated as one route by which subpicomolar concentrations of nanoparticles may be readily detected and sized,^189^ the secondary advantage of this methodology is the minimisation of problems associated with nanoparticle adsorption onto the electrode support.

**Figure 30:**
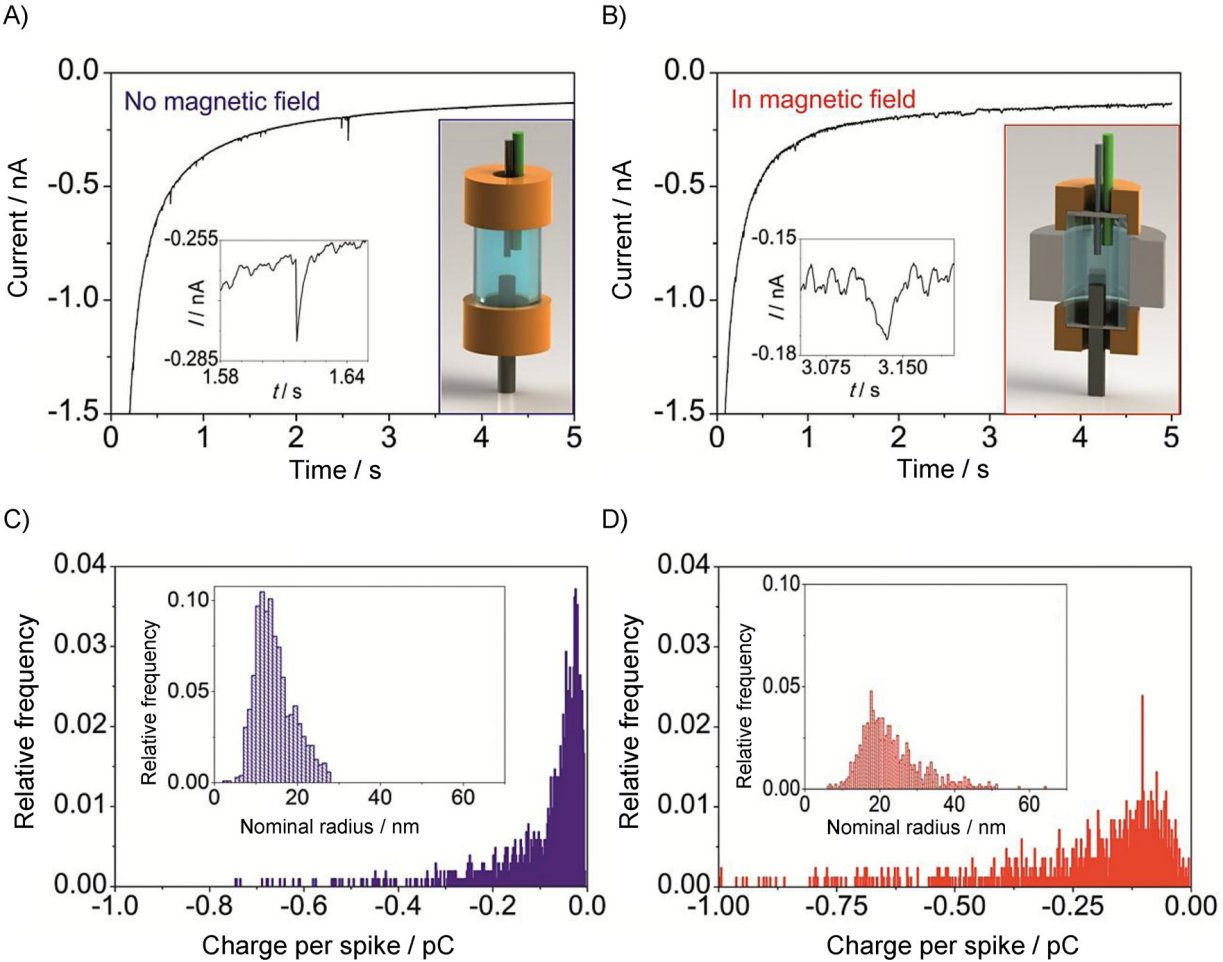
Chronoamperograms showing reductive impact spikes recorded without (A) and in (B) a magnetic field and the derived impact charge and NP size distributions (C and D); electrolyte: 0.2 m phosphate buffer (pH 10), E (vs. SCE)=0.9 V. Reproduced with permission from Ref. 191 Copyright 2014, PCCP Owner Societies.

Beyond being an analytical technique, the direct nano-impact method also provides a route for investigating more fundamental problems. A first example is the use of the technique for the study and monitoring of solution-phase nanoparticle agglomeration and aggregation.^190^ In a similar vein, the magnetic-field-induced agglomeration of Fe_3_O_4_ has also been directly evidenced.^191^ Figure 30 depicts examples of iron oxide reduction spikes and the associated size distributions obtained in the presence and absence of a magnetic field. Second, the electron-transfer kinetics and mechanism to the nanoparticles can be studied.183b,^192^ Third, the interaction of the nanoparticle with the electrochemical double layer is of utmost importance and the nano-impacts methodology has provided a direct route by which these interactions can be probed.^193^

Consequently, we conclude that the use of ‘mediated’ and ‘direct’ nano-impact experiments have significant potential for future research both as nano-metrology techniques and as novel methods for addressing fundamental and technological nanoparticle challenges.

## Conclusion

The above survey shows that with the era of numerical simulation, the technique of voltammetry has come of age and has power to contribute very significantly, not least in a quantitative manner, to many problems in analytical, physical, and biophysical chemistry, as well as to nanochemistry and nanotechnology. Further rapid advances are anticipated.
